# Quantum spin coherence and electron spin distribution channels in vanadyl-containing lantern complexes†

**DOI:** 10.1039/d3qi01806g

**Published:** 2023-11-03

**Authors:** Manuel Imperato, Alessio Nicolini, Marco Borsari, Matteo Briganti, Mario Chiesa, Yu-Kai Liao, Antonio Ranieri, Arsen Raza, Enrico Salvadori, Lorenzo Sorace, Andrea Cornia

**Affiliations:** a Dipartimento di Scienze Chimiche e Geologiche e UdR INSTM, Università degli Studi di Modena e Reggio Emilia via G. Campi 103 41125 Modena Italy acornia@unimore.it; b Dipartimento di Scienze Fisiche, Informatiche e Matematiche, Università degli Studi di Modena e Reggio Emilia via G. Campi 213/A 41125 Modena Italy; c Dipartimento di Chimica “Ugo Schiff” e UdR INSTM, Università degli Studi di Firenze via della Lastruccia 3 50019 Sesto Fiorentino FI Italy; d Dipartimento di Chimica e NIS Centre, Università degli Studi di Torino via P. Giuria 7 10125 Torino Italy; e Dipartimento di Scienze della Vita, Università degli Studi di Modena e Reggio Emilia via G. Campi 103 41125 Modena Italy

## Abstract

We herein investigate the heterobimetallic lantern complexes [PtVO(SOCR)_4_] as charge neutral electronic qubits based on vanadyl complexes (*S* = 1/2) with nuclear spin-free donor atoms. The derivatives with R = Me (1) and Ph (2) give highly resolved X-band EPR spectra in frozen CH_2_Cl_2_/toluene solution, which evidence the usual hyperfine coupling with the ^51^V nucleus (*I* = 7/2) and an additional superhyperfine interaction with the *I* = 1/2 nucleus of the ^195^Pt isotope (natural abundance *ca.* 34%). DFT calculations ascribe the spin density delocalization on the Pt^2+^ ion to a combination of π and *δ* pathways, with the former representing the predominant channel. Spin relaxation measurements in frozen CD_2_Cl_2_/toluene-*d*_8_ solution between 90 and 10 K yield *T*_m_ values (1–6 μs in 1 and 2–11 μs in 2) which compare favorably with those of known vanadyl-based qubits in similar matrices. Coherent spin manipulations indeed prove possible at 70 K, as shown by the observation of Rabi oscillations in nutation experiments. The results indicate that the heavy Group 10 metal ion is not detrimental to the coherence properties of the vanadyl moiety and that Pt–VO lanterns can be used as robust spin-coherent building blocks in materials science and quantum technologies.

## Abstract 2

Lantern complexes containing VO^2+^ and Pt^2+^ ions show electron spin coherence with phase memory times above 10 μs at 10 K. EPR spectra prove the delocalization of the unpaired spin density to the heavy metal ion through both π and *δ* coupling channels.

## Graphical abstract

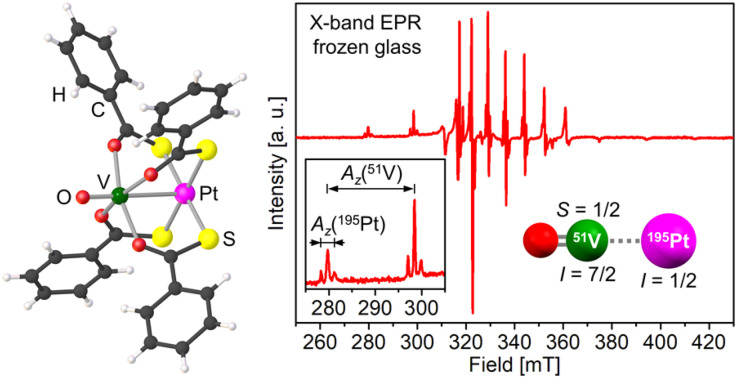

## Introduction

In the past 15 years, paramagnetic molecular complexes have been actively investigated as promising platforms for implementing quantum technologies.^1–4^ This followed the discovery that the coherence time of their electronic spin, *T*_m_ (*i.e.*, the lifetime of the spin superposition state), can be long enough to allow spin manipulation by using pulsed magnetic resonance techniques.^5^ Compared to other investigated architectures, the molecular approach provides the possibility of fine-tuning the chemical and physical properties of the qubit by rational chemical design.^6–8^ In addition to allowing control over the sources of spin decoherence, this permits engineering the inter-spin interactions necessary to build universal quantum gates^9–13^ and endowing molecules with the features necessary for their controlled organization on a surface.^14–17^

Among the molecules investigated as potential spin qubits, those containing the vanadyl ion (VO^2+^) have been reported to show almost invariably good coherence times^18–23^ which persist up to room temperature.^19,24,25^ Furthermore, thanks to the *I* = 7/2 nuclear spin of ^51^V, whose natural abundance (NA) is as high as 99.75%, vanadium(iv) complexes can also be operated as nuclear qudits with an electronic spin ancilla.^26–28^

Lantern complexes, also known as paddlewheel structures, are among the most versatile and robust building blocks in metal–organic chemistry. Their application potential covers catalysis, biomedical sciences, molecular recognition, and the assembly of molecular frameworks.^29–33^ Depending on the presence of either three or four bridging ligands (typically carboxylates^34^), they come in two main variants: trigonal and tetragonal, and often contain metal–metal bonds.^35,36^ The choice of the constituent metal ions is virtually limitless and has afforded a multitude of homo- and heterobimetallic variants. Examples of paramagnetic paddlewheels are numerous,^37,38^ but those incorporating vanadyl ions are comparatively rare. Available examples are limited to heterobimetallic species containing a heavy metal ion like Pd^2+^ or Pt^2+^ and (N,S), (N,O), or (O,S) bridging ligands like pyridine-2-thiolates,^39^ 2-pyridonates,^40^ and monothiocarboxylates.^41^ These ligands coordinate following hard–soft Lewis acid–base principles,^42^ that is, any O donor is bonded to VO^2+^, whereas any S donor coordinates the Group 10 metal ion.

We herein investigate monothiocarboxylato-bridged lanterns [PtVO(SOCR)_4_] with R = Me (1) and Ph (2)^41^ as a new class of qubits exhibiting a combination of attractive features, which are otherwise separately present in different families of molecular spin qubits: (i) the V^4+^ ion is only coordinated by O donors, which are virtually nuclear spin-free; (ii) the ligand's skeleton comprises only S, C, and H atoms, and the nuclear spins are primarily embedded in the latter; (iii) the chemical structure can afford fourfold molecular symmetry, thereby simplifying the magnetic and spectroscopic responses; (iv) these lanterns are neutral and potentially processable materials, *e.g.* by vapor phase methods, and the PtS_4_ moiety may act as a decoupling layer between the VO^2+^ complex and a surface; (v) the superhyperfine interaction of the unpaired electron with the nucleus of ^195^Pt (*I* = 1/2, NA = 33.83%) may allow for the direct measurement of the degree of spin delocalization on the PtS_4_ moiety.

We find that the highly resolved X-band EPR spectra exhibited by these complexes in frozen solution allow for the accurate measurement of the superhyperfine coupling with ^195^Pt, in addition to the usual hyperfine interaction with the ^51^V nucleus. We then use DFT calculations to ascertain the origin of the superhyperfine coupling with ^195^Pt. Quantum spin coherence is detected by pulsed EPR spectroscopy up to 90 K, and coherent spin manipulations at 70 K are demonstrated by the Rabi oscillations observed in nutation experiments.

## Results and discussion

Compounds 1 and 2·CH_2_Cl_2_ were prepared in pure crystalline form as described by Doerrer *et al.*^41^ Note that 2·CH_2_Cl_2_ loses lattice solvent rapidly, so that the actual composition of dry samples is 2·*x*CH_2_Cl_2_ with *x* < 1 (see the ESI†). However, for the sake of simplicity, the compound will be indicated as 2·CH_2_Cl_2_ throughout. Characterization data in the solid state by combustion analysis, single-crystal X-ray diffraction, and IR spectroscopy were found to be consistent with those reported by the above authors (see the ESI†).

**Fig. 1:**
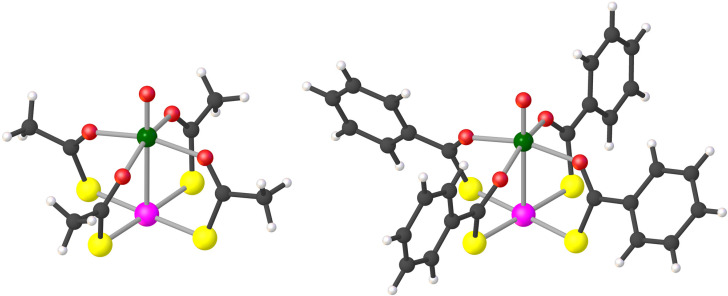
Molecular structure of paddlewheel complexes in 1 (left) and 2·CH_2_Cl_2_ (right). Color code: dark gray = C, light gray = H, red = O, yellow = S, green = V, and pink = Pt. Selected distances and angles: V=O = 1.58–1.59 Å, V–O = 1.99–2.02 Å, O=V–O = 98.1–99.3°, O–V–O = 87.9–90.9°, Pt–S = 2.32 Å, and S–Pt–S = 88.8–90.9°. The line connecting V and Pt does not represent a chemical bond.^41^

The structure of the lantern complexes in 1 and 2·CH_2_Cl_2_, as obtained by single-crystal X-ray diffraction at 100 K, is depicted in Fig. 1 (ellipsoid plots are available in Fig. S1†). The two metal centers are at 2.8635(6) Å and 2.7823(10) Å from each other, respectively. All O donors are bonded to the V^4+^ ion, which achieves a square-pyramidal coordination geometry, whereas the four S donors provide a square-planar environment around the Pt^2+^ ion. The crystallographic molecular symmetry is only C_1_, but the two paddlewheels approach fourfold symmetry quite closely in the solid state. Importantly, they assemble into dimers (V⋯V = 8.9–9.0 Å), although with a different interaction mode in the two compounds (Fig. S2†). In 1, two approximately coaxial and pseudo-staggered molecules form a C_2_-symmetric dimer *via* a metallophilic Pt⋯Pt interaction [3.1747(4) Å, S–Pt⋯Pt–S = 32.2–33.1°]. On the other hand, paddlewheel dimers in 2·CH_2_Cl_2_ are centrosymmetric and comprise two offset molecules interacting through a pair of Pt⋯S contacts [3.1266(14) Å]. It must be noted that a Pt–VO paddlewheel compound supported by 4-methylpyridine-2-thiolates assembles into one-dimensional chains *via* V=O⋯Pt contacts rather than forming dimers.^39^

We first extended to 2·CH_2_Cl_2_ the magnetic characterization in the solid state, reported in ref. 41 only for 1. The high-temperature value of *χ*_M_*T*, where *χ*_M_ is the magnetic susceptibility per mole of vanadyl units and *T* is temperature, is 0.365 emu K mol^−1^ and agrees with the expectations for isolated *S* = 1/2 centers with *g* slightly smaller than 2.00 (0.368 emu K mol^−1^ for *g* = 1.98) (Fig. S6†). The *χ*_M_*T* product decreases significantly upon cooling below 30 K, suggesting the presence of weak antiferromagnetic coupling between vanadyl ions. In Fig. S6†, we also present isothermal molar magnetization *vs*. field data for 2·CH_2_Cl_2_ at three different temperatures (1.9, 2.5, and 4.5 K). At the lowest temperature, the highest measured magnetization value is close to 1.0 *μ*_B_ per vanadyl moiety. A simultaneous fit of these magnetization and susceptibility data was performed using spin Hamiltonian (SH)^43^*Ĥ* = *J***Ŝ**_1_· **Ŝ**_2_ + *gμ*_B_(**Ŝ**_1_ + **Ŝ**_2_)·**B** with *S*_1_ = *S*_2_ = 1/2 and expressing the calculated values per vanadyl moiety. When *g* was fixed at 1.975, a value typical of vanadyl complexes, *J* = 0.644(2) cm^−1^ was obtained as best-fit value (Fig. S6†).^44^ A significantly stronger antiferromagnetic interaction (*J* = 4.70 cm^−1^) was reported for 1.^41^

Finally, since vanadyl complexes often display slow relaxation of magnetization in applied fields, we also performed alternating-current magnetic susceptibility measurements on both compounds (see the ESI and Fig. S7†). However, we did not observe any imaginary component of the susceptibility, indicating that the observed antiferromagnetic coupling hampers slow relaxation of magnetization in the solid state.

**Fig. 2:**
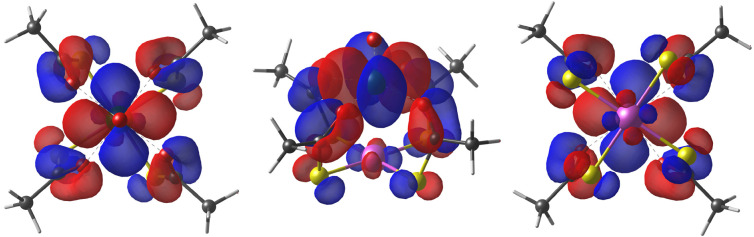
Top, side, and bottom views of the DFT-computed singly occupied quasi-restricted orbital (QRO) of 1. The isosurface value is set to 0.013 e^−^*a*_0_^−3^ (an additional plot at 0.05 e^−^*a*_0_^−3^ is available in Fig. S10†). The color code is the same as in Fig. 1.

As first observed by Doerrer *et al.*,^41^ the V⋯V distances between adjacent molecular pairs in the crystal are as short as 4.89 Å in 1 and 4.77 Å in 2·CH_2_Cl_2_. Therefore, the involved superexchange coupling pathway is not necessarily intra-dimer in nature. DFT calculations were thus performed to determine the electronic structure of individual paddlewheels and unveil the mechanism of magnetic interaction among them in the solid state (see the ESI†). Single paddlewheels feature significant spin delocalization from V^4+^ to Pt^2+^*via δ* and π interactions (Fig. 2). The *δ* interaction occurs directly through space and arises from an efficient overlap between the 3d_*xy*_ and 5d_*xy*_ orbitals of V and Pt, respectively. The π interaction is mediated by the π system of the monothiocarboxylato ligands, whose O 2pπ and S 3pπ orbitals efficiently interact with the 3d_*xy*_ and 5d_*xy*_ orbitals of V and Pt, respectively. The resulting Löwdin spin density on Pt is 0.014 e^−^ in 1 and 0.011 e^−^ in 2. These values exceed those found on monothiocarboxylato O atoms directly bonded to V^4+^ and are consistent with the superhyperfine interaction with ^195^Pt observed in the EPR spectra (*vide infra*).

Further calculations on dimer models within the broken symmetry (BS) approach showed that intra-dimer coupling in 1 is antiferromagnetic with *J* = 6.55 cm^−1^, whereas inter-dimer interaction is much weaker (−0.290 cm^−1^). A much smaller antiferromagnetic interaction is predicted within the dimers of 2·CH_2_Cl_2_ (*J* = 0.466 cm^−1^), again surpassing in magnitude inter-dimer coupling (0.130 cm^−1^). A comparison with the experimental *J* values (4.70 cm^−1^ in 1^41^ and 0.64 cm^−1^ in 2·CH_2_Cl_2_) indicates that intra-dimer interaction is the leading coupling mechanism in both compounds. Furthermore, calculations reproduce well the smaller *J* value measured in thiobenzoato derivative 2·CH_2_Cl_2_. Since the extent of spin delocalization on Pt is comparable in the two compounds, we ascribe the observed trend to geometrical factors. In 1, the neighboring Pt^2+^ ions are indeed only 3.175 Å apart from each other and lie along the pseudo-C_4_ axis of the dimer. The significant deviation of the average S–Pt⋯Pt–S dihedral angle (32.6°) from the staggered value (45°) then allows for some overlap between the magnetic orbitals of the two paddlewheels through the Pt 5d_*xy*_ component. However, the two neighboring Pt^2+^ ions in 2·CH_2_Cl_2_ are not on the same axis but are engaged in Pt⋯S contacts, which results in a much weaker interaction.

The behavior of 1 and 2 in organic solvents was studied by a manifold of techniques before EPR experiments. The authors of ref. 41 quote room-temperature *μ*_eff_ values measured by the Evans method as a proof of the occurrence of monomeric complexes in CD_2_Cl_2_. We contend that these values would be insensitive to the weak superexchange coupling observed within molecular pairs, as evidenced by the solid-state magnetic susceptibility measured at room temperature. Direct estimation of molecular weight (MW) in solution was then attempted by ^1^H NMR diffusion ordered spectroscopy (DOSY), whose successful application to paramagnetic compounds of first-row transition metals has only recently been demonstrated.^45^ While 1 yielded featureless ^1^H NMR spectra in CD_2_Cl_2_, 2 afforded one well-resolved resonance assigned to the *p*-Ar protons (*δ* = 8.50 ppm, Δ*ν*_½_ = 89 Hz), which proved usable for a ^1^H NMR DOSY experiment (Fig. S3 and S4†). A procedure based on external calibration curves (ECCs, see the ESI for details†) afforded MW = 560 ± 170, 630 ± 200, or 620 ± 280 g mol^−1^ depending on the particular ECC used (Table S1†).^45,46^ Considering that the DOSY technique tends to underestimate the MW of solutes with high molecular densities,^45,47^ the results provide a strong indication that 2 is monomeric in CD_2_Cl_2_ (expected MW = 810.7 g mol^−1^).

Cyclic voltammetry was also performed on 1 mM solutions of 1 and 2 in CH_2_Cl_2_ at −23 °C (Fig. S5†). Both paddlewheels show a well-defined, quasi-reversible one-electron reduction/oxidation process at a formal potential *E*°′ = −1.737 V in 1 and −1.506 V in 2 (*vs.* Fc^+^/Fc). The thiobenzoato derivative 2 displays an additional cathodic signal at a more negative potential, lacking the anodic counterpart, which is indicative of an irreversible reduction process. The peak currents of all the signals are proportional to the square root of the potential scan rate, indicating diffusion-controlled electron transfer processes. The above-quoted formal potential value correlates well with the energy of the LUMO (−1.813 eV in 1 and −2.116 eV in 2), which provides the electron affinity within the Koopmans’ theorem (after sign reversal). Therefore, the larger reduction potential observed for 1 agrees with its computed lower electron affinity.

To gain additional insight into this redox process, one-electron reduced 1 (1^−^) was simulated by DFT and its structure was optimized in a vacuum. The paddlewheel structure is perfectly preserved upon reduction, and the final geometry is almost superimposable on the neutral one. More interestingly, the extra-electron is located on the 5d_*x*^2^−*y*^2^_ orbital of Pt, while the occupation of the 3d orbitals of V is unmodified. This 5d_*x*^2^−*y*^2^_ orbital shows an extensive delocalization on S atoms (Fig. S12†), as expected from its antibonding character, and some electron density is also present on V. Therefore, the reduction process leaves the V center unaffected, and a biradical species is formed with Pt showing +1 oxidation state.

**Fig. 3:**
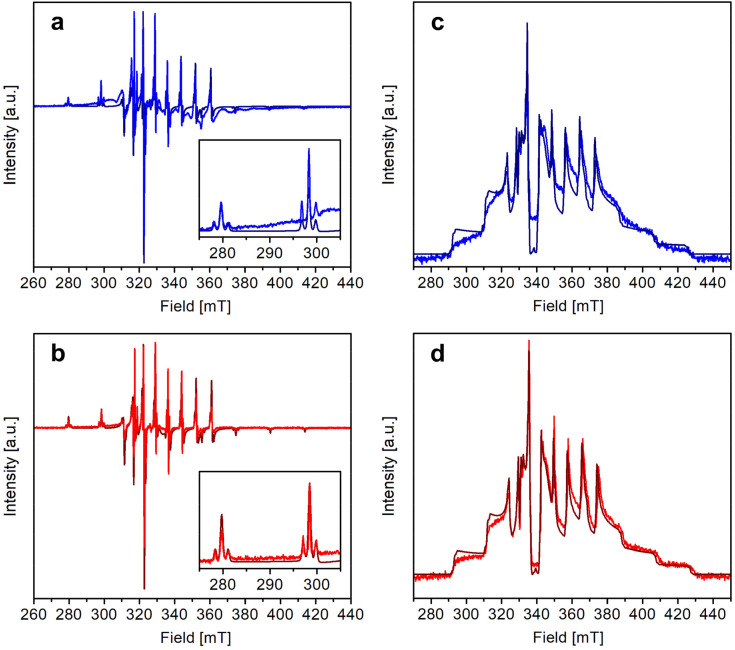
X-band CW- (a and b) and ESE-detected EPR spectra (c and d) of 1 (a and c) and 2 (b and d). Experimental conditions: (a and b) 4 mM in CH_2_Cl_2_/toluene (1 : 1 v/v), *ν* ≅ 9.40 GHz, and *T* = 30 K; (c and d) 1 mM in CD_2_Cl_2_/toluene-*d*_8_ (1 : 1 v/v), *ν* ≅ 9.74 GHz, and *T* = 10 K. The experimental/simulated spectra are drawn in blue/navy (a and c) or in red/wine (b and d) for 1 and 2, respectively.

The X-band continuous wave (CW)- and electron spin echo (ESE)-detected EPR spectra of 1 and 2 were recorded in CH_2_Cl_2_/toluene or CD_2_Cl_2_/toluene-*d*_8_ (1 : 1 v/v) frozen solution at 1–4 mM vanadyl concentration. The experimental spectra, shown in Fig. 3, are typical of isolated axial vanadyl moieties (*S* = 1/2), the broad background observed in the CW spectra of 1 being attributable to residual clustered species (expanded views of the spectra are available in Fig. S8†). The spectra are dominated by the anisotropic hyperfine coupling of the electron spin with the nuclear spin of ^51^V (*I* = 7/2), which splits each resonance, affording an eight-line pattern. Interestingly, the very narrow linewidth of the CW-EPR spectra allows us to clearly distinguish the further splitting of the signals due to the superhyperfine coupling with the nuclear spin of ^195^Pt (*I* = 1/2, NA = 33.83%), which produces satellite signals on either side of the main EPR lines. A superhyperfine coupling with Pt, although partly obscured by the broad linewidth of the spectrum, was previously reported in *S* = 1/2 paddlewheel [ClPtNi(OH_2_)(SOCPh)_4_].^48^ However, to the best of our knowledge, its observation in molecules containing VO^2+^ is unprecedented.

Following this interpretation, all experimental spectra were simulated^49^ using the SH in eqn (1):1
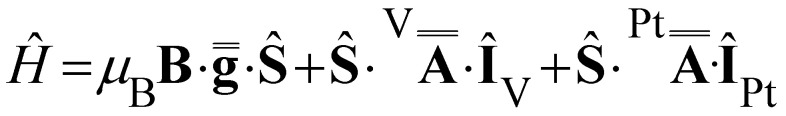
where *S* = 1/2, *I*_V_ = 7/2, *I*_Pt_ = 1/2 for ^195^Pt, and *I*_Pt_ = 0 for the remaining isotopes of Pt (the NA of the different isotopes was included in the calculations). The 
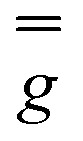
 matrix and the hyperfine 
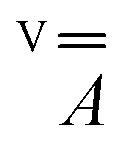
 and 
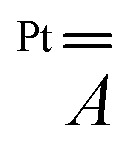
 tensors were assumed to be axial and collinear. The SH parameters that provide the best simulation of the experimental CW-EPR spectra are gathered in Table 1 and the corresponding calculated spectra are shown in Fig. 3 and Fig. S8.† The principal components of 
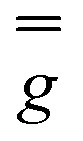
 and 
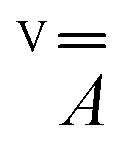
 are fully consistent with those of other axial vanadyl complexes, with the unpaired electron mainly localized in the 3d_*xy*_ orbital.^18^ It is worth stressing that the two complexes have very close SH parameters, further indicating that their structure is similar and that the structural differences observed in the crystalline state are mostly lost in frozen solution.

**Table 1:** SH parameters for 1 and 2 from EPR spectra and DFT calculations^a^

	1		2	
	EPR	DFT	EPR	DFT
*g*_*x*,*y*_	1.991(1)	1.991	1.990(1)	1.988, 1.991
*g*_*z*_	1.9350(5)	1.950	1.9338(3)	1.952
^V^ *A*_*x*,*y*_	196.0(5)	−268.33	197.0(5)	−262.310, −265.532
^V^ *A*_*z*_	517.5(3)	−594.06	518.5(3)	−587.888
^Pt^ *A*_*x*,*y*_	47(2)	−14.332, −14.247	42(2)	−11.596, −12.625
^Pt^ *A*_*z*_	80.0(5)	−41.741	74.0(5)	−36.856

a ^V,Pt^*A*_*x*,*y*,*z*_ values are given in MHz. Experimental ^V,Pt^*A*_*x*,*y*,*z*_ parameters are absolute values.

In particular, the quality of the simulations shows that any rhombic distortion is below experimental resolution and that both complexes approach axial symmetry. The spectra also firmly exclude intermolecular magnetic couplings comparable to those detected in the solid state (*J* ∼ 1–5 cm^−1^) and prove that 1 and 2 exist as monomers in organic solution. As the most significant difference between the two derivatives, the superhyperfine coupling with ^195^Pt is slightly larger in 1 than in 2 [^Pt^*A*_*z*_ = 80.0(5) *vs.* 74.0(5) MHz; ^Pt^*A*_*x*,*y*_ = 47(2) *vs.* 42(2) MHz]. These values correspond however to a similar spin density delocalized on Pt, which is estimated to be of the order of 2.7% for both complexes (see the ESI†).

The SH parameters were simulated by DFT on the isolated molecules optimized in a vacuum (see the ESI†). The values so obtained are in fair agreement with those extracted from EPR spectra (Table 1).

The hyperfine coupling is slightly overestimated for V and underestimated for Pt, but the overall trend is nicely reproduced. A larger rhombicity is computed for 2, whose optimized structure exhibits a more pronounced deviation from C_4_ symmetry. The superhyperfine coupling with ^195^Pt is correctly predicted to be larger for 1 than for 2. To gain insight into the origin of this trend, in Table S5† we present the computed contributions to the 
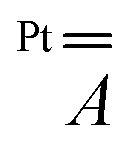
 tensor. The modulation mainly arises from a reduced Fermi contact term, and this happens notwithstanding the shorter Pt⋯V distance in the optimized structure of 2 (Table S4†). Moreover, when the optimized structure of 2 is modified by replacing Ph with Me substituents, the computed superhyperfine coupling with Pt is practically unaffected. This shows that the smaller Fermi contact term in 2 is due to different geometrical parameters rather than to the electron-withdrawing effect of the Ph substituents. Specifically, the average V–O⋯S–Pt dihedral angle varies from 22.7° in 1 to 24.7° in 2 (Table S4†). These values are somewhat larger than those observed in the solid state (17.1 and 22.5°, respectively) but exhibit the same trend in the two derivatives. The larger dihedral angles of the thiobenzoato derivative then reduce the spin delocalization (*vide supra*) through π bonding, which would be maximized for dihedral angles close to zero. The importance of the π interaction through the ligands is further confirmed by model 1_*δ*_, where the π channel is switched off and the two metals can communicate only *via δ* interaction (see the ESI and Fig. S11†). The computed Fermi contact contribution to 
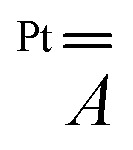
 is reduced (in absolute value) from 19.843 MHz in 1 to 5.638 MHz in 1_*δ*_ (Table S5†). We infer that the *δ* interaction accounts for roughly one-third of the spin delocalization on the Pt^2+^ ion, while the main channel is the π interaction through the thioacetato ligands.

Finally, DFT calculations indicate that the large anisotropy of the 
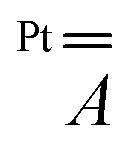
 tensor mainly arises from the spin–orbit interaction of the Pt^2+^ ion, while the dipolar component plays a minor role (Table S5†).

**Fig. 4:**
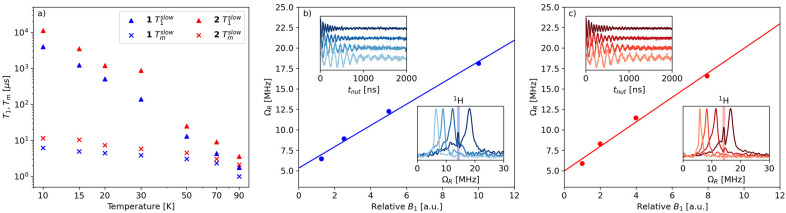
(a) Temperature dependence of *T*_1_ (triangles) and *T*_m_ (crosses) for 1 (blue) and 2 (red). Only the slow component of the bi-exponential fit is plotted. (b and c) Linear dependence of the Rabi frequency (*Ω*_R_) as a function of the relative intensity of the oscillating field *B*_1_ in 1 (b) and 2 (c) at 70 K. The insets of panels (b) and (c) display Rabi oscillations measured at different microwave attenuation (upper left) and the Fourier transform of the Rabi oscillations (lower right), with the shaded areas at *ca.* 14 MHz denoting the Larmor frequency of weakly coupled ^1^H nuclei. All spectra were measured at the maximum echo intensity on 1 mM frozen solutions in CD_2_Cl_2_/toluene-*d*_8_ (1 : 1 v/v).

The spin relaxation properties of 1 and 2 were studied by pulsed-EPR spectroscopy at X-band frequency. Temperature-dependent inversion recovery and echo decay experiments were used to determine the spin–lattice relaxation time (*T*_1_) and the coherence time (*T*_m_), respectively, in the temperature interval between 10 and 90 K (Fig. 4a). The samples were dissolved in deuterated solvents and measured as frozen solutions at a magnetic field setting corresponding to the maximum intensity of ESE-detected EPR spectrum (Fig. 3c and d). *T*_1_ and *T*_m_ values were extracted by fitting the experimental inversion recovery and echo decay traces, respectively (Fig. S9† and Table S2†).

The temperature dependence of *T*_m_ for 1 (Fig. 4a) is modest between 10 K and 70 K, with values in the range 6–2 μs and a drop at 90 K (*T*_m_ = 1 μs). Complex 2 displays slightly longer relaxation times than 1, with values in the range 11–2 μs between 10 K and 90 K. We note that the sudden decrease in *T*_m_ observed in 1 at 90 K is consistent with the discontinuity displayed by complexes [Cu(Et_2_dtc)_2_] and [Cu(Et_2_dtp)_2_] in the temperature range 85–130 K, which was attributed to the effect of methyl rotations (Et_2_dtc^−^ = diethyldithiocarbamate, Et_2_dtp^−^ = diethyldithiophosphate).^50^ Moreover, phenyl substituents in a similar complex, [Cu(Ph_2_dtp)_2_] (Ph_2_dtp^−^ = diphenyldithiophosphate), were shown to affect *T*_m_ in a higher temperature range (120–250 K).^50^

The *T*_m_ values of 1 and 2 compare favorably with those of known vanadyl-based qubits in frozen solutions, which are typically in the 1–20 μs range and strongly depend on the presence of protonated solvents.^18,20–23,28^*T*_m_ values of 4–6 μs were reported in protonated solvents,^20^ while *T*_m_ can reach up to 20 μs in deuterated solvents.^21–23^ An exceptionally long *T*_m_ (>100 μs) was reported for bis-dithiolene vanadyl complexes in SO_2_, a nuclear spin-free solvent.^21^ This comparison indicates that the orbital overlap between V and Pt is not detrimental to the coherence properties of the vanadyl complex, despite the large spin–orbit coupling constant of Pt (almost fourty times larger than the corresponding value for V).^51^

To demonstrate the possibility of coherent spin manipulations, *i.e.*, placing the spins in any arbitrary superposition of states, nutation experiments were performed at different microwave powers at 70 K. Rabi oscillations were clearly observed with the expected linear dependence of the Rabi frequency (*Ω*_R_) as a function of the microwave attenuation (Fig. 4b and c).

For both samples, the electronic spin–lattice relaxation time *T*_1_ was measured in the 10–90 K temperature range. Fig. 4a shows the slow component of the bi-exponential fit, which is usually taken to represent the spin–lattice relaxation (*T*_1_). The fast component, associated with spectral diffusion effects (*i.e.*, excitation bandwidth smaller than the spectral width), is not considered.

Similar to *T*_m_, a longer *T*_1_ was observed for 2, with a maximum value of *ca.* 11 ms at 10 K and the lowest value of 4 μs at 90 K. *T*_1_/*T*_m_ data were however not recorded above 90 K because of the echo intensity decay related to the onset of molecular libration as the melting point of the solvent is approached. The temperature dependence of *T*_1_ could be modelled considering a direct mechanism of relaxation at low temperatures and a local vibrational mode responsible for high-temperature relaxation,^26,52^ according to eqn (2):2
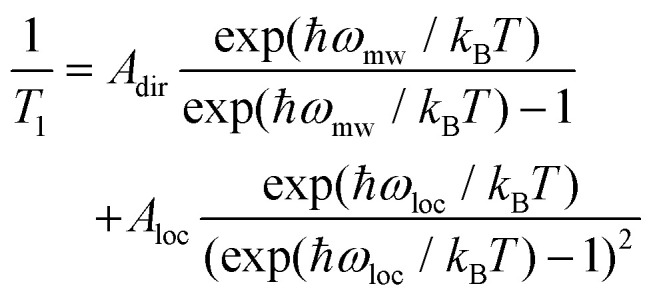


**Fig. 5:**
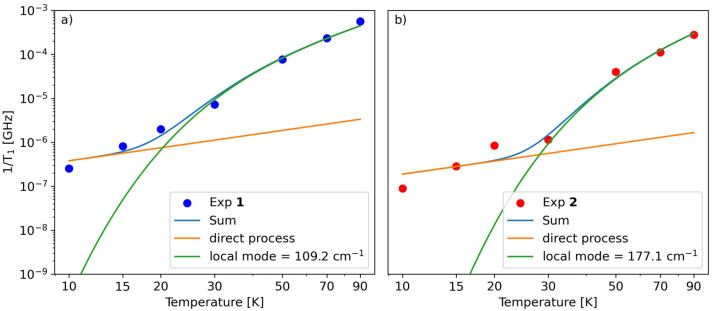
Fit of the dependence of *T*_1_*vs.* temperature for 1 (a) and 2 (b) using eqn (2).

The first term in eqn (2) represents the direct mode (*ω*_mw_ is the microwave frequency, with *ω*_mw_/2π = 9.74 GHz), while the second term accounts for a Raman process promoted by an optical mode of frequency *ω*_loc_, which represents an average of the effective vibrations. This model fairly reproduces the temperature dependence of *T*_1_ for both samples, providing a mean frequency with *ħω*_loc_ = 109 cm^−1^ and 177 cm^−1^ for 1 and 2, respectively (Fig. 5 and Table S3†). While these values are in line with estimates recently reported for other vanadium-based molecular qubits,^26^ lower vibrational modes in the order of tens of cm^−1^ have been identified as drivers of the longitudinal relaxation in similar systems.^26,53^

A theoretical analysis of the normal vibration modes of 1 and 2 lends considerable support to the above results (Tables S6 and S7†). Consistent with the average values obtained from the fitting, normal modes that affect the coordination sphere of V^4+^ are expected at frequencies below 200 cm^−1^ in both compounds, with the lowest around 60–70 cm^−1^.^54^ However, another key parameter is the symmetry of the normal vibration modes, which determines the magnitude of the spin-phonon coupling, as demonstrated elsewhere.^55–57^ For complexes with C_4_ symmetry, the normal modes belonging to the totally symmetric irreducible representation are the ones exerting the largest coupling and governing the spin–lattice relaxation.^56^ Although the two paddlewheels only have approximate C_4_ symmetry, by inspection of displacement vectors in the first coordination sphere it is straightforward to individuate those modes that would be totally symmetric in exact C_4_ symmetry. This analysis showed the presence of totally symmetric vibrations located at 104 cm^−1^ in 1 and at 171 cm^−1^ in 2, *i.e.* very close to the values resulting from the fitting (Tables S6, S7 and Fig. S13†). Although the effect of the solvent was not included in the DFT simulation, the agreement is remarkably good and shows again the predictive power of such a chemically intuitive approach. Moreover, in both cases, large contributions to the modes arise from the oxygen atoms in the first coordination sphere, as expected, but also from the methyl and phenyl groups. This feature could suggest a fine-tuning of the relaxation properties by acting on the substituents of the monothiocarboxylato ligands.

## Conclusions

Lantern complexes containing VO^2+^ and Pt^2+^ ions bridged by monothiocarboxylates, [PtVO(SOCR)_4_], are a new family of electrically neutral molecular qubits where the paramagnetic vanadyl ion (*S* = 1/2) is situated in a uniaxial coordination environment with nuclear spin-free donors. The hyperfine interaction with the ^51^V nucleus (*I* = 7/2) is accompanied by a further measurable superhyperfine coupling with the fraction of ^195^Pt nuclei (*I* = 1/2) precisely located on the fourfold molecular axis. This additional interaction is triggered by the nonnegligible spin density transferred to the Pt^2+^ ion *via* π and *δ* pathways over a V⋯Pt distance of 2.8–2.9 Å. As a result, the ^195^Pt-^51^V isotopomer contains one electronic qubit (*S* = 1/2) coupled with a nuclear qudit (*I* = 7/2) and with a nuclear qubit (*I* = 1/2). Rewardingly, the spin coherence properties in frozen organic solution compete with those of the best-known vanadyl-based qubits, with *T*_m_ values reaching up to 11 μs for the derivative with R = Ph embedded in a CD_2_Cl_2_/toluene-*d*_8_ matrix at 10 K. The possibility of a coherent manipulation of the electronic spin was indeed demonstrated by the observation of Rabi oscillations in nutation experiments.

The Group 10 metal ion and its square-planar coordination geometry offer attractive perspectives for functionalization and processing. The assembly of paddlewheels into dimers in the solid state through either metallophilic interactions or Pt⋯S contacts indicates a significant tendency of the heavy metal ion to engage in additional bonding interactions. For instance, the flat and electronically “soft” PtS_4_ moiety may offer a viable route to chemisorb these lanterns on metal surfaces. The fixed Pt⋯V distance of 2.8–2.9 Å is expected to ensure a weak electronic coupling between the VO^2+^ ion and the surface, with no need for a decoupling layer. Furthermore, unlike with VOPc^58,59^ and titanium sandwich compounds,^60^ paddlewheel molecules are expected to adopt a unique orientation on the surface, namely with the V=O vector pointing away from the surface. Attempts in this direction are underway in our laboratories.

## Author contributions

Manuel Imperato: investigation, validation, visualization, and writing – review & editing. Alessio Nicolini: formal analysis, investigation, validation, visualization, and writing – review & editing. Marco Borsari: formal analysis, investigation, validation, and writing – review & editing. Matteo Briganti: data curation, formal analysis, investigation, software, visualization, and writing – review & editing. Mario Chiesa: data curation, formal analysis, funding acquisition, resources, visualization, and writing – review & editing. Yu-Kai Liao: investigation and writing – review & editing. Antonio Ranieri: data curation, formal analysis, investigation, validation, visualization, and writing – review & editing. Arsen Raza: formal analysis, investigation, validation, visualization, and writing – review & editing. Enrico Salvadori: writing – review & editing. Lorenzo Sorace: data curation, formal analysis, investigation, resources, validation, visualization, and writing – review & editing. Andrea Cornia: conceptualization, funding acquisition, methodology, project administration, resources, supervision, and writing – original draft.

## Conflicts of interest

There are no conflicts to declare.

## Supplementary Material

QI-011-D3QI01806G-s001

QI-011-D3QI01806G-s002

QI-011-D3QI01806G-s003

QI-011-D3QI01806G-s004
