# Supplementary material for: Quantum spin coherence and electron spin distribution channels in vanadyl-containing lantern complexes
Source: Inorg Chem Front. 2023 Nov 3;11(1):186–95. doi: 10.1039/d3qi01806g (PMC10782212; doi:10.1039/d3qi01806g)
Supplement: QI-011-D3QI01806G-s001 [file QI-011-D3QI01806G-s001.pdf]

Electronic Supplementary Material (ESI) for Inorganic Chemistry Frontiers.
This journal is © the Partner Organisations 2023

Quantum spin coherence and electron spin distribution channels in vanadyl-containing lantern complexes

Manuel Imperato, Alessio Nicolini, Marco Borsari, Matteo Briganti, Mario Chiesa, Yu-Kai Liao, Antonio Ranieri, Arsen Raza, Enrico Salvadori, Lorenzo Sorace, and Andrea Cornia*

Abstract: We herein investigate the heterobimetallic lantern complexes [PtVO(SOCR)$_4$] as electrically neutral electronic qubits based on vanadyl complexes (S = 1/2) with nuclear spin-free donor atoms. The derivatives with R = Me (**1**) and Ph (**2**) give highly resolved X-band EPR spectra in frozen CH_2Cl_2/toluene solution, which evidence the usual hyperfine coupling with the ^{51}V nucleus (I = 7/2) and an additional superhyperfine interaction with the I = 1/2 nucleus of the ^{195}Pt isotope (natural abundance ca. 34%). DFT calculations ascribe the spin-density delocalization on the Pt^{2+} ion to a combination of π and δ pathways, with the former representing the predominant channel. Spin relaxation measurements in frozen CD_2Cl_2/toluene-d_8 solution between 90 and 10 K yield T_m values (1-6 μs in **1** and 2-11 μs in **2**) which compare favorably with those of known vanadyl-based qubits in similar matrices. Coherent spin manipulations indeed prove possible at 70 K, as shown by the observation of Rabi oscillations in nutation experiments. The results indicate that the heavy Group 10 metal ion is not detrimental to the coherence properties of the vanadyl moiety and that Pt-VO lanterns can be used as robust spin-coherent building blocks in materials science and quantum technologies.

Electronic Supplementary Information

Experimental Procedures

General Methods

Synthesis of [PtVO(SOCMe)$_4$] (1)

Synthesis of [PtVO(SOCPh)$_4$]·CH$_2$Cl$_2$ (2·CH$_2$Cl$_2$)

Additional structural diagrams

Fig. S1. Structure of paddlewheel complexes in **1** and **2**·CH$_2$Cl$_2$ at 100 K with displacement ellipsoids

Fig. S2. Structure of paddlewheel dimers in **1** and **2**·CH$_2$Cl$_2$ at 100 K

Further details on NMR spectroscopy

Fig. S3. ^{1}H NMR spectrum of **2**·CH$_2$Cl$_2$ in CD$_2$Cl$_2$

Fig. S4. ^{1}H NMR DOSY spectrum of **2**·CH$_2$Cl$_2$ in CD$_2$Cl$_2$

Table S1. ^{1}H NMR DOSY data for **2**·CH$_2$Cl$_2$ in CD$_2$Cl$_2$

Additional electrochemical data

Fig. S5. Cyclic voltammograms of **1** and **2** as 1 mM solutions in CH$_2$Cl$_2$ at T = −23 °C

Details on magnetic measurements

Fig. S6. Dc magnetic data for **2**·CH$_2$Cl$_2$

Fig. S7. Field dependence of ac magnetic susceptibility for **1** and **2**·CH$_2$Cl$_2$ at 1.9 K

Details on EPR spectroscopy

Fig. S8. Expanded views of the perpendicular and parallel regions of the spectra in Fig. 3a,b

Determination of the spin-density distribution on Pt from ^{195}Pt hyperfine couplings

T_1 and T_m vs temperature data

Fig. S9. Inversion recovery and echo decay traces for **1** and **2**

Table S2. T_1 and T_m fitting parameters for **1** and **2**

Table S3. Best-fit parameters in eqn (2) for **1** and **2**

Computational Procedures

Computational details

Fig. S10. DFT computed singly occupied Quasi-Restricted Orbital (QRO) of **1** and **2** with an isosurface value set to 0.05 e$^-$ a$_o$$^{-3}$

Fig. S11. Geometrical model **1**$_\delta$ employed to compute the π and δ contributions to the Fermi contact term

Fig. S12. Platinum-centered singly occupied Quasi-Restricted Orbital (QRO) of the anion **1**$^-$, as computed by DFT

Table S4. Main structural parameters of the optimized structures of **1** and **2** at the DFT level of theory

Table S5. Contributions to the ^{195}Pt hyperfine coupling tensor for **1**, **2**, and **1**$_\delta$ as computed by DFT

Table S6. Calculated vibrational frequencies for **1**

Table S7. Calculated vibrational frequencies for **2**

Fig. S13. Displacement vectors of selected vibrational modes in **1** and **2**

References

Experimental Procedures

General methods. Nondeuterated solvents were either purchased anhydrous (CH_2Cl_2) or dried using standard methods (toluene, *n*-hexane, $CHCl_3$),[1] whereas CD_2Cl_2 (99.8%D) and toluene-d_8 (99.5%D) were used as received. Both nondeuterated and deuterated solvents were deoxygenated through three *freeze−pump−thaw* cycles and stored over activated 4 Å molecular sieves in a nitrogen-filled MBraun UniLAB glove box. Tetra-*n*-butylammonium chloride (TBACl) was purified as described elsewhere.[2] The amount of crystallization water in commercial $VOSO_4 \cdot xH_2O$ was determined by combustion analysis (x = 3.6). Compounds **1** and **2**·CH_2Cl_2 were prepared in pure crystalline form following the procedure described by Doerrer *et al.*,[3] with only minor modifications (see below).

Combustion analysis was performed using a ThermoFisher Scientific Flash 2000 analyzer. IR spectra were collected in ATR mode on a JASCO 4700 FT-IR spectrometer, between 400 and 4000 cm^{-1} and with a resolution of 2 cm^{-1}. Electronic spectra were collected in CH_2Cl_2 using a Jasco V-570 UV-Vis-NIR spectrometer operating in double-beam mode (optical path length l = 0.1 cm). ^1H NMR spectroscopy measurements were conducted in CD_2Cl_2 at 298 K on an AVANCE400 FT-NMR spectrometer from Bruker Biospin (400.13 MHz), using 5 mm airtight Young-valved NMR tubes from Norell. Spectra were analyzed using TopSpin (version 4.2.0).[4] The chemical shifts (δ) are expressed in ppm downfield from tetramethylsilane (TMS) as internal standard. The following abbreviations are used in reporting spectroscopic data: s, singlet; br, broad; sh, shoulder.

A Bruker-Nonius X8APEX machine, equipped with a Mo Kα generator and an area detector, was used for single-crystal X-ray diffraction studies at 298 K. The crystals were soaked in NHV immersion oil (Jena Bioscience), mounted on a MiTeGen Microloop, and measured.

A potentiostat/galvanostat PARSTAT mod. 2273A (EG&G PAR, Oak Ridge, USA) was used for cyclic voltammetry measurements. Experiments were carried out at different scan rates (v = 0.02-5 V s^{-1}) using a cell for small volume samples (2 mL). A 1-mm diameter glassy carbon (GC) disk (PAR), a Pt ring, and an Ag wire were used as working, counter, and quasi-reference electrodes, respectively. The GC electrode was cleaned following a previously reported procedure.[2,5] All measurements were made in a nitrogen-filled MBraun UniLAB glovebox at −23 °C, using 1 mM solutions of the complexes in CH_2Cl_2 and 0.05 M TBACl as supporting electrolyte. The ohmic drop between the working and the reference electrodes was minimized through a careful feedback correction. The ferrocenium/ferrocene (Fc^+/Fc) redox couple (in CH_2Cl_2, $E°'$ = 0.460 V *vs.* SCE) was used to calibrate the potential of the quasi-reference electrode.[6] The formal potential value ($E°'$) of the electron transfer processes was calculated as the semi-sum of the cathodic and anodic peak potentials, $E°' = E_{1/2} = (E_{pc}+E_{pa})/2$. All the reported $E°'$ values are referred to the Fc^+/Fc redox couple. The experiments were repeated at least five times and $E°'$ values were found to be reproducible within ±0.002 V.

Synthesis of [PtVO(SOCMe)$_4$] (1). We closely followed the procedure described by Doerrer *et al.*, working with the same quantities of reactants.[3] However, we observed that the addition of the K_2PtCl_4 solution to the solution of sodium thioacetate ($NaHCO_3$ + CH_3COSH) gave a cloudy orange mixture rather than a pink solution; after overnight stirring, the mixture turned into a clear solution of orange rather than yellow color. After the addition of $VOSO_4 \cdot 3.6H_2O$, the crude product was obtained as a light green powder (192.7 mg, 69% referred to Pt) analyzing as **1**·0.2$VOSO_4$. Elemental analysis calcd (%) for **1**·0.2$VOSO_4$: C 16.15, H 2.03; found: C 16.06, H 1.92. Layering *n*-hexane over a $CHCl_3$ saturated solution of the crude product (149.7 mg in ~5 mL) gave X-ray quality green blocks of **1** after approximately two weeks. Crystals were separated and dried under mild vacuum (101.9 mg, 72% crystallization yield).

IR spectrum is consistent with that reported;[3] UV-Vis-NIR (CH_2Cl_2, $1.42 \cdot 10^{-2}$ M): λ_{max} (ε) ~400 (sh), 616 (19), 731 nm (22 M^{-1} cm^{-1}); elemental analysis calcd (%) for **1**: C 17.08, H 2.15; found: C 17.15, H 1.97; multiple crystals of **1** were checked by single-crystal X-ray diffraction, which afforded unit cell metrics and parameters consistent with published data.[3]

Synthesis of [PtVO(SOCPh)$_4$]·CH_2Cl_2 (2·CH_2Cl_2). The literature synthetic route to compound **2**·CH_2Cl_2[3] was slightly modified, as we replaced potassium thiobenzoate with a solution of thiobenzoic acid and $NaHCO_3$, as proposed by Dahl *et al.* for the synthesis of a similar nickel compound.[7] In a 25 mL round bottom flask, $NaHCO_3$ (65.8 mg, 0.783 mmol) was dissolved in 10 mL of H_2O to yield a colorless solution. PhCOSH (85.0 µL, 0.726 mmol) was added to this solution, which turned light yellow after ~5 minutes. The mixture was allowed to react for 30 minutes until evolution of CO_2 ceased. It was then decanted away from any trace of unreacted acid (yellow drops) and transferred to a clean 25 mL flask. K_2PtCl_4

(75.5 mg, 0.182 mmol) and $VOSO_4 \cdot 3.6H_2O$ (41.1 mg, 0.180 mmol) were separately dissolved in 1.5 mL and 2 mL of H_2O, respectively. The K_2PtCl_4 solution was added to the sodium thiobenzoate solution and the resulting dark orange suspension was allowed to react for 1 hour, yielding a light orange color. Dropwise addition of the vanadyl sulfate solution to the mixture resulted in the immediate formation of a light green precipitate. Reaction was continued overnight, then the solid was collected by filtration using a fritted glass funnel (porosity G4) and extensively washed with H_2O. This crude product was dried overnight under vacuum, yielding a green-yellow powder (154.5 mg, 99.7% referred to V) analyzing as **2**·0.35PhCOSH. Elemental analysis calcd (%) for **2**·0.35PhCOSH: C 42.57, H 2.59; found: C 42.58, H 2.56. Layering of *n*-hexane over a CH_2Cl_2 saturated solution of the crude product (130.5 mg in ~7 mL) gave X-ray quality green blocks of **2**·CH_2Cl_2 after approximately two weeks. Crystals were separated and stored under the mother liquor. Before any characterization, the mother liquor was decanted away from the crystals, which were then dried under N_2 flush. Notice that the elemental composition reported by Doerrer *et al.* indicates complete loss of lattice solvent from the sample.[3] We found that even freshly isolated crystals analyze as **2**·$x$$CH_2Cl_2$ with $x < 1$ and that progressive loss of crystallization solvent is unavoidable upon drying. All characterization data reported hereafter were taken on samples with accurately known dichloromethane content (x). For the sake of simplicity, however, the compound will be indicated as **2**·CH_2Cl_2 throughout.

^1H NMR (400.13 MHz, CD_2Cl_2, 298 K): δ = 8.50 (s, 4H; *p*-Ar), 4.4 (br s, 8H; *m*-Ar), 5.33 ppm (s, 2H; CH_2Cl_2, ~0.5 mol per mole of **2**); IR spectrum is consistent with that reported;[3] UV-Vis-NIR (CH_2Cl_2, $8.31 \cdot 10^{-3}$ M): λ_{max} (ε) ~430 (sh), 620 (39), 727 nm (42 M^{-1} cm^{-1}); elemental analysis calcd (%) for **2**·0.65CH_2Cl_2: C 39.74, H 2.48; found (on freshly isolated crystals): C 39.70, H 2.55; elemental analysis calcd (%) for **2**·0.4CH_2Cl_2: C 40.38, H 2.48; found (on crystals kept in a sealed vial for 1 month): C 40.32, H 2.39; multiple freshly isolated crystals of **2**·CH_2Cl_2 were checked by single-crystal X-ray diffraction, which afforded unit cell metrics and parameters consistent with published data.[3]

Additional structural diagrams

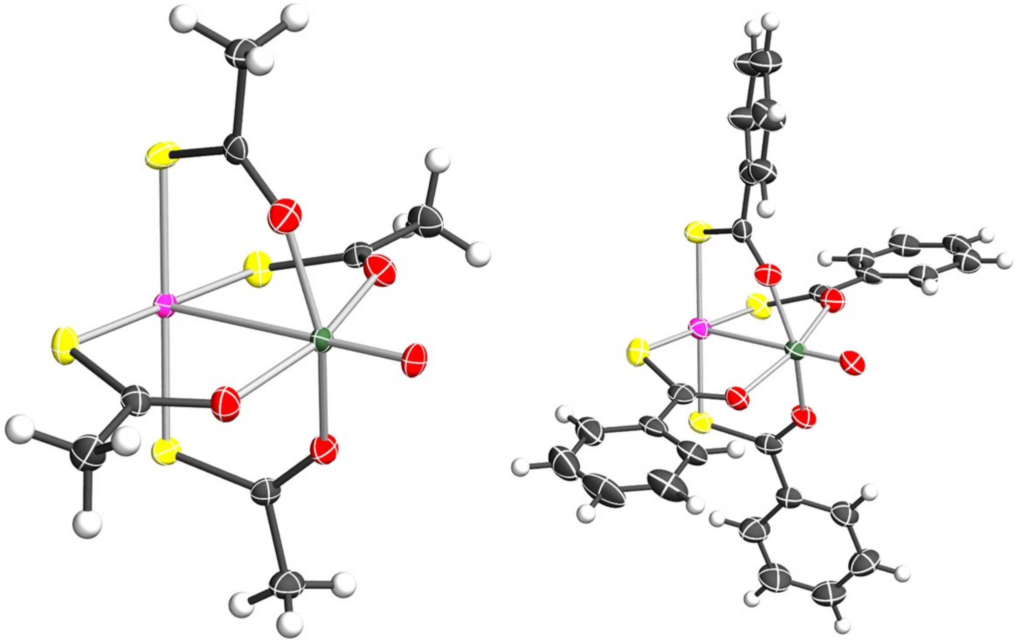

Fig. S1. Structure of paddlewheel complexes in **1** (left) and **2**·CH_2Cl_2 (right) at 100 K with displacement ellipsoids drawn at the 80% probability level. Color code: dark gray = C, light gray = H, red = O, yellow = S, green = V, pink = Pt. The line connecting V and Pt does not represent a chemical bond. From Ref.[3]

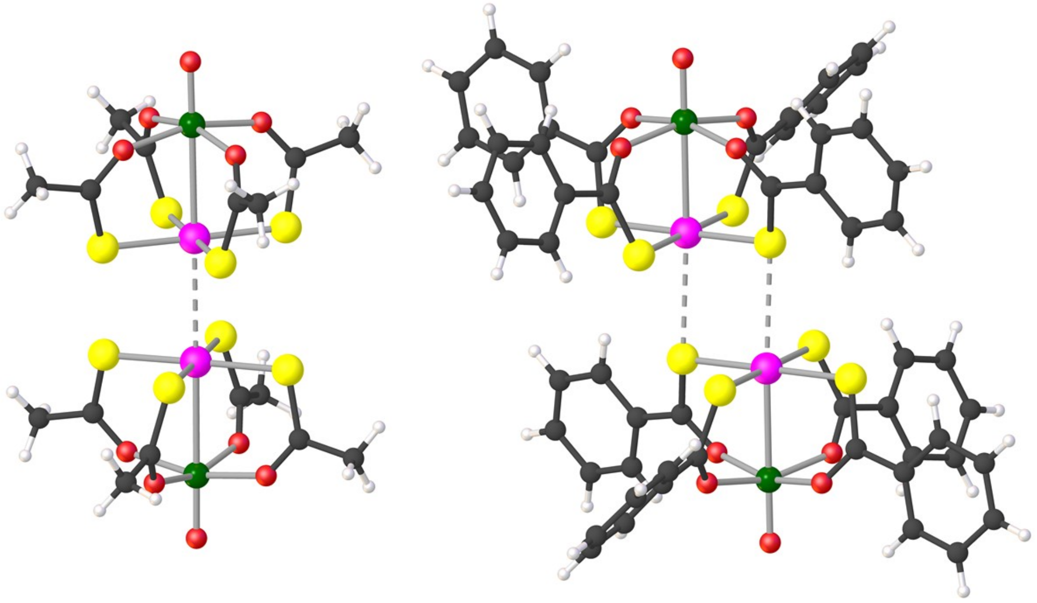

Fig. S2. Structure of paddlewheel dimers in **1** (left) and **2**·CH_2Cl_2 (right) at 100 K. Color code is the same as in Fig. S1. **1** forms C_2-symmetric dimers in the solid state via metallophilic Pt···Pt interactions [3.1747(4) Å, S–Pt···Pt–S = 32.2-33.1°], while **2** forms square centrosymmetric dimers where two offset molecules interact through a pair of Pt···S contacts [3.1266(14) Å]. Intradimer V···V separation is 8.90 Å in **1** and 8.95 Å in **2**·CH_2Cl_2. The line connecting V and Pt does not represent a chemical bond. From Ref.[3]

Further details on NMR spectroscopy. The samples investigated by ^{1}H NMR spectroscopy were prepared inside a nitrogen-filled MBraun UniLAB glove box by charging 5 mm airtight Young-valved NMR tubes with 1.8 and 4.2 mg of crystalline **1** and **2**·CH_2Cl_2, respectively, dissolved in ~0.5 mL of CD_2Cl_2. **1** was found NMR silent, most likely because of the proximity of methyl groups to the paramagnetic center. **2**·CH_2Cl_2 (Fig. S3) gave a broad singlet at 8.50 ppm ($\Delta\nu_{1/2}$ = 89 Hz) and a much broader feature at 4.4 ppm ($\Delta\nu_{1/2}$ ~ 900 Hz). An attempt to deconvolute the signals despite the unfavorable linewidth gave a 1:2.43 integrated intensity ratio. This suggests assigning the two peaks to the *p*-Ar and *m*-Ar protons of the thiobenzoato ligands, respectively, the *o*-Ar peak being presumably too broad to be detectable. After 50-fold dilution of the solution in CD_2Cl_2, the *p*-Ar signal was still well visible at the same chemical shift and no new peaks appeared in the aromatic region. Thus, no ligand dissociation is detected within the sensitivity of ^{1}H NMR spectroscopy. Working on the more concentrated solution, the *p*-Ar resonance gave a measurable signal in a DOSY experiment using the *ledbpgp2s* sequence (Bruker library), with a diffusion time (d20) *big delta* of Δ = 0.013 s, and a gradient length (P30) *small delta* of δ = 1700 μs (the DOSY spectrum is shown in Fig. S4). State-of-the-art external calibration curves (ECCs) for small organic molecules were used to determine MW in CD_2Cl_2, with TMS as an internal reference compound for the normalization of diffusion coefficients (D), according to the procedure described by Stalke *et al.*[8] An additional calibration curve developed by Byers *et al.*[9] for species containing 3d metals was also tested, using CH_2Cl_2 and $CDHCl_2$ as internal references to normalize the measured D. The results are presented in Table S1. The absolute errors on estimated MWs were calculated as reported by Stalke *et al.*,[10] who showed that these errors are largely determined by the uncertainties on ECC's parameters.

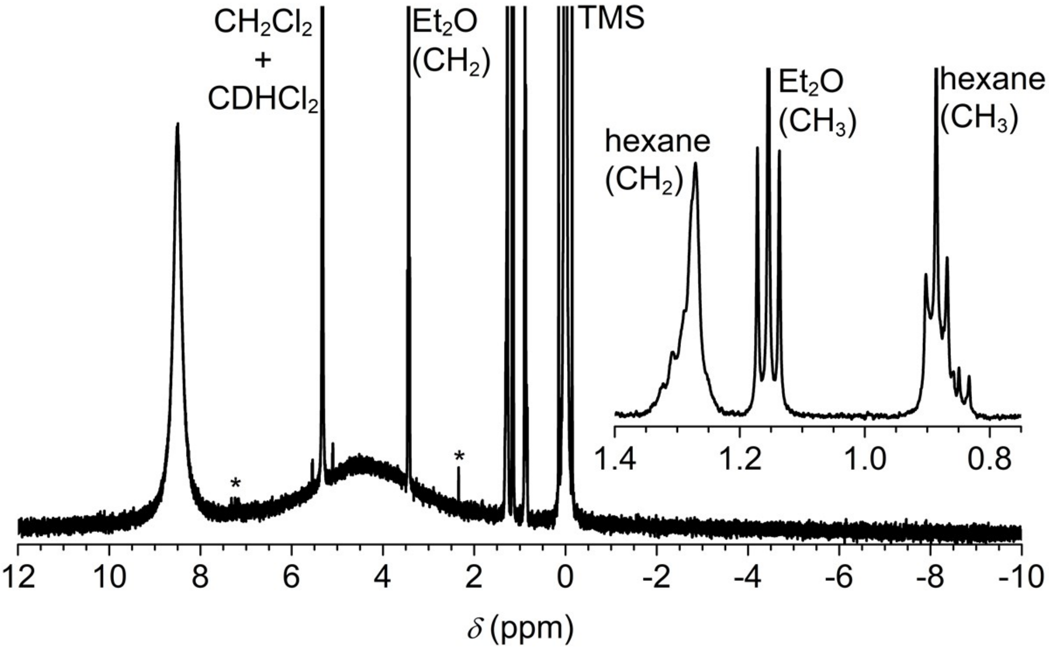

Fig. S3. ^{1}H NMR spectrum of **2**·CH_2Cl_2 in CD_2Cl_2 (0.01 M, 298 K, 400.13 MHz). TMS was added as an internal standard for DOSY experiments. Et_2O and toluene (*) are contaminants from the glove box atmosphere, while *n*-hexane is a residual from crystallization. Processing parameters (TopSpin 4.2.0[4]): SI = TD, LB = 0.30 Hz.

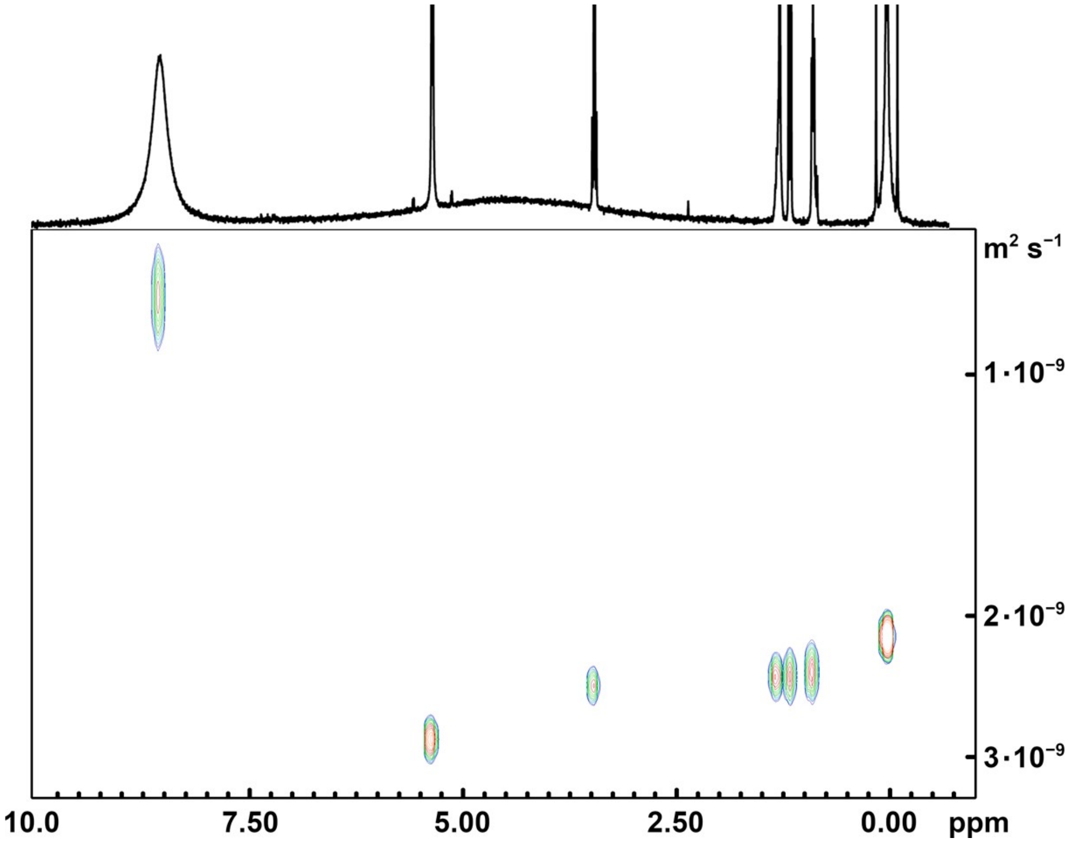

Fig. S4. ^{1}H NMR DOSY spectrum of **2**$\cdot CH_2Cl_2$ in CD_2Cl_2 (0.01 M, 298 K, 400.13 MHz).

Table S1. ^{1}H NMR DOSY data for **2**$\cdot CH_2Cl_2$ in CD_2Cl_2.[a]

ECC	$\log D_x$ [b]	$\log D_{ref,fix}$ [c]	$\log D_{ref}$ [d]	$\log D_{x,norm}$ [e]	MW (g mol^{-1})
DSE [f]	−9.10	−8.6253	−8.67	−9.05	563±169
MCC [g]					628±205
Byers *et al.* [h]		−8.46	−8.55	−9.01	623±276

[a] See Ref.[8], unless otherwise noted. [b] Measured *D* of **2**. [c] Tabulated *D* of the internal reference. [d] Measured *D* of the internal reference. [e] Normalized diffusion coefficient of **2**, obtained as $\log D_{x,norm} = \log D_{ref,fix} - \log D_{ref} + \log D_x$. [f] Dissipated spheres and ellipsoids. [g] Merged calibration curve. [h] Calibration curve for species containing 3d metals from Ref.[9]

Additional electrochemical data

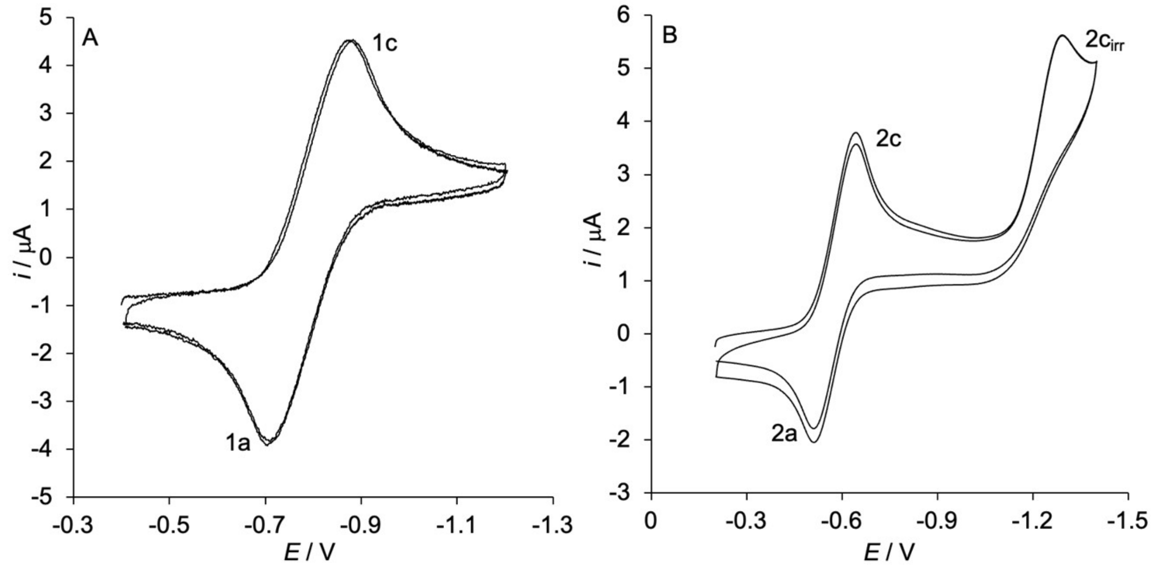

Fig. S5. Cyclic voltammograms of **1** (A) and **2** (B) as 1 mM solutions in CH_2Cl_2 at $T = −23$ °C. GC working electrode, Ag wire quasi-reference electrode, 0.05 M TBACl supporting electrolyte, scan rate $v = 0.05$ V s^{-1}. 1c/1a and 2c/2a mark the quasi-reversible cathodic/anodic peaks, while $2c_{irr}$ is the irreversible cathodic peak of **2**.

Details on magnetic measurements. The direct-current (dc) measurements (Fig. S6) were performed on a microcrystalline sample (22.25 mg) analyzing as **2**$\cdot 0.3CH_2Cl_2$ and wrapped in Teflon tape (4.00 mg) using a Quantum Design Magnetic Properties Measuring System (QD-MPMS) equipped with a superconductive quantum interference device (SQUID). Low-field magnetization (M) data were recorded at $H = 10$ kOe between 30 and 289 K and at $H = 1$ kOe between 1.9 and 50 K. Magnetic susceptibility was then calculated as $\chi = M/H$. Isothermal magnetization measurements were also performed at 1.9, 2.5, and 4.5 K in applied fields up to 50 kOe. Molar susceptibility (χ_M) and magnetization (M_M) data were obtained by using the molar mass (836.22 g mol^{-1}) and intrinsic diamagnetism ($-388.78 \cdot 10^{-6}$ emu mol^{-1}, estimated from Pascal's constants[11]) appropriate for **2**$\cdot 0.3CH_2Cl_2$, and correcting for the diamagnetic contribution of Teflon ($-3.7 \cdot 10^{-7}$ emu g^{-1}). The alternating-current (ac) susceptibility measurements (Fig. S7) were performed on samples of **1** (9.89 mg, 562.46 g mol^{-1}) and **2**$\cdot CH_2Cl_2$ (analyzing as **2**$\cdot 0.3CH_2Cl_2$; 10.97 mg, 836.22 g mol^{-1}) using the same instrument in an interval of frequencies between 1 and 1000 Hz, and in fields from 0 to 10000 Oe.

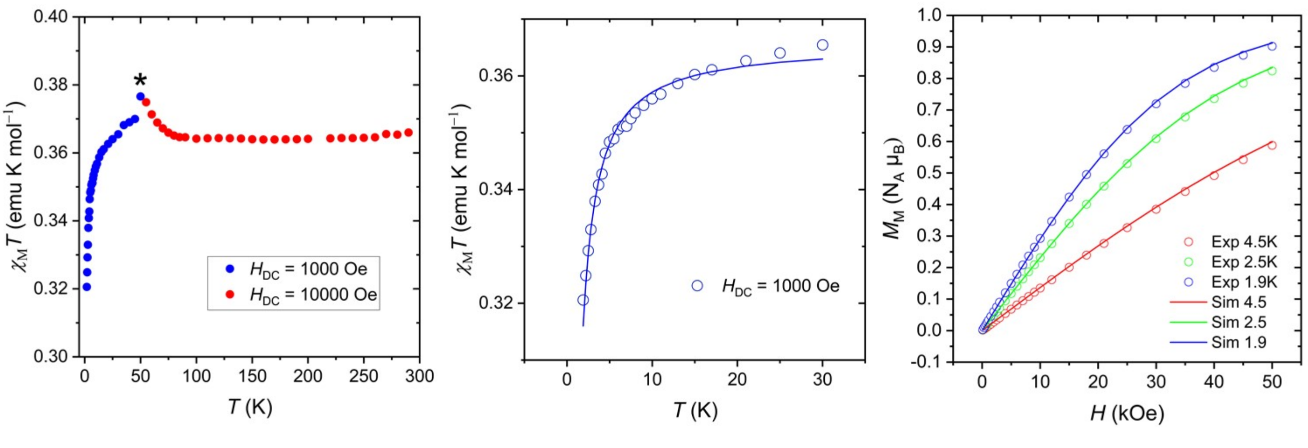

Fig. S6. Dc magnetic data for **2**$\cdot CH_2Cl_2$. (Left) $\chi_M T$ vs T plot at two different fields (1 and 10 kOe). The asterisk evidences a feature corresponding to the onset of antiferromagnetic transition of residual molecular oxygen present in the sample chamber. (Center) $\chi_M T$ vs T data from 1.9 to 30 K at 1 kOe. (Right) Isothermal magnetization curves at three different temperatures as a function of applied field. The solid lines are the best-fit simulations obtained with the parameters reported in the text.

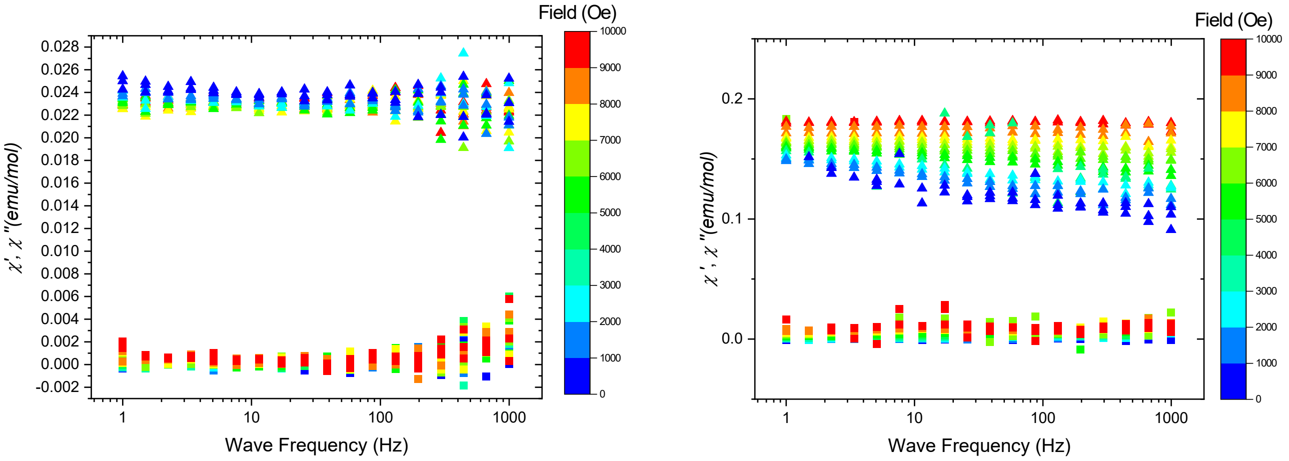

Fig. S7. Field dependence of ac magnetic susceptibility [$\chi'(\omega)$, triangles; $\chi''(\omega)$, squares] for **1** (left) and **2**·CH_2Cl_2 (right) at 1.9 K.

Details on EPR spectroscopy. X-band ($\nu \cong$ 9.40 GHz) CW-EPR spectra were recorded on a Bruker Elexsys E500 spectrometer equipped with an SHQ cavity. Low temperature measurements (30 K) were obtained using an Oxford Instruments ESR900 continuous flow helium cryostat and temperature controlled by an Oxford Instruments ITC503. Solutions of **1** and **2**·CH_2Cl_2 (4 mM) in CH_2Cl_2/toluene (1:1 v/v) were prepared in a nitrogen-filled glove box with oxygen and water levels below 0.5 ppm. The EPR tubes were sealed with a home-made rubber cap, extracted from the glove box, and rapidly cooled down to N_2 boiling temperature just before the measurements. X-band ($\nu \cong$ 9.74 GHz) pulsed-EPR spectra were recorded on a Bruker Elexsys E580 spectrometer equipped with a dielectric ring resonator (ER 4118X-MD5) housed in a Cryogenic Cryogen-free variable temperature cryostat. Solutions of **1** and **2**·CH_2Cl_2 (1 mM) in CD_2Cl_2/toluene-d_8 (1:1 v/v) were prepared in an argon-filled glove box with oxygen and water levels below 0.5 ppm. The EPR tubes were sealed with PTFE tape, extracted from the glove box, and rapidly cooled down to N_2 boiling temperature just before the measurements. During the measurements, the resonator was over-coupled to minimize ringdown following the application of the microwave pulses. ESE-detected EPR spectra were measured at 10 K using a Hahn echo sequence ($\pi/2 - \tau - \pi - \tau - echo$) while sweeping the field with τ = 200 ns. Coherence times were measured using the Hahn echo sequence with incremented τ. Spin lattice relaxation times were measured using inversion recovery sequence $\pi - t_w - \pi/2 - \tau - \pi - \tau - echo$ with incremented waiting time t_w and τ = 200 ns. Rabi oscillations were measured using sequence $t_{nut} - t_w - \pi/2 - \tau - \pi - \tau - echo$ by incrementing the length of the nutation pulse t_{nut} varying microwave power attenuation from 0 to 10 dB and fixing t_w = 3 μs and τ = 200 ns. The nutation pulse length varied depending on the microwave attenuation.

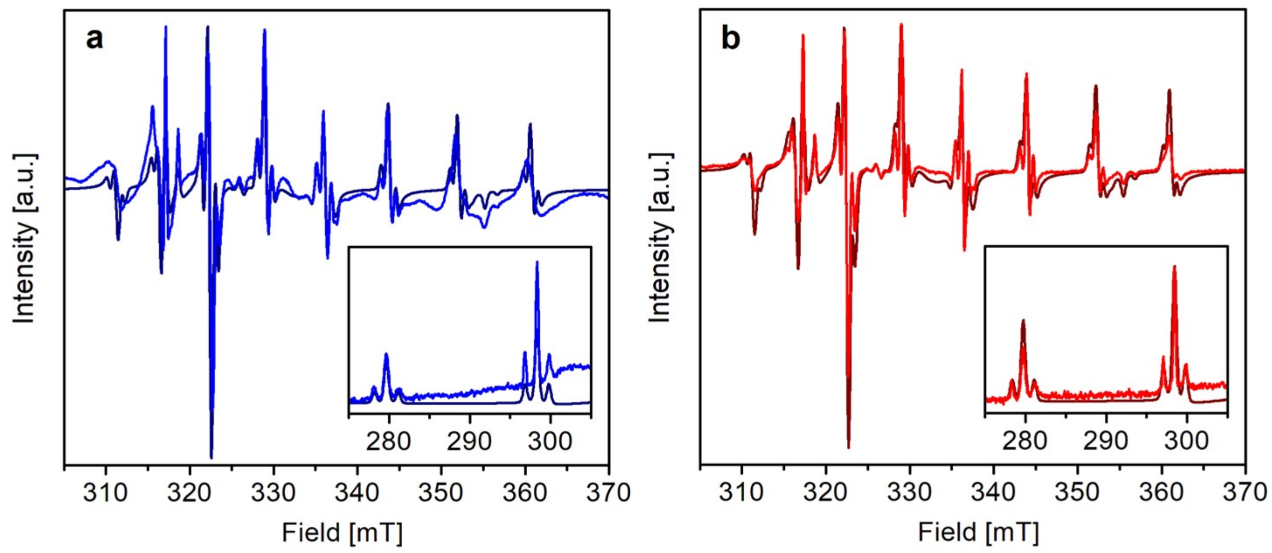

Fig. S8. Expanded views of the perpendicular and parallel (inset) regions of the spectra in Fig. 3a (a) and Fig. 3b (b). Color code is the same as in Fig. 3.

Determination of the spin-density distribution on Pt from ^{195}Pt hyperfine couplings. From the observation of the hyperfine structure and determination of the isotropic (a_{iso}) and dipolar (T) hyperfine couplings, the electronic spin distribution in a molecular fragment can be obtained. With the knowledge of a_{iso} and T for the atomic species (a_0 and T_0), and assuming that the hyperfine interaction at a given nucleus is proportional to the electron spin density at that nucleus, one can obtain the spin population in the s-type (ρ_s) and d-type (ρ_d) orbitals of Pt. Although such relationships are very approximate, they are nevertheless useful and almost universally applied to interpret EPR data. Assuming that the matrix elements have the same sign, in accordance with DFT results, the experimental ^{195}Pt hyperfine matrices can be decomposed into the isotropic and dipolar values (in MHz) as follows:

1: $$^{Pt}\bar{\bar{A}} = \begin{pmatrix} 47 & 0 & 0 \\ 0 & 47 & 0 \\ 0 & 0 & 80 \end{pmatrix} = 58\bar{\bar{I}} + \begin{pmatrix} -11 & 0 & 0 \\ 0 & -11 & 0 \\ 0 & 0 & 22 \end{pmatrix} \quad \text{(S1a)}$$

2: $$^{Pt}\bar{\bar{A}} = \begin{pmatrix} 42 & 0 & 0 \\ 0 & 42 & 0 \\ 0 & 0 & 74 \end{pmatrix} = 52.6\bar{\bar{I}} + \begin{pmatrix} -10.6 & 0 & 0 \\ 0 & -10.6 & 0 \\ 0 & 0 & 21.2 \end{pmatrix} \quad \text{(S1b)}$$

where $\bar{\bar{I}}$ is the identity matrix. For an unpaired electron (free electron, g_e = 2.0023) on a ^{195}Pt nucleus with a unitary spin population in an s-type orbital (ρ_s = 1), one would observe an isotropic hyperfine coupling constant of a_0 = 34410 MHz. If the electron resides in a d-type orbital one would observe a uniaxial hyperfine constant of T_0 = –421 MHz.[12] Including a correction for the difference in the g values (g_{iso} = 1.97), the spin populations in s-type and d-type orbitals can thus be estimated as in eqn (S2):

$$\rho_s = \frac{a_{iso}\, g_e}{a_0\, g_{iso}} \qquad \rho_d = \frac{T\ g_e}{T_0 g_{iso}} \quad \text{(S2)}$$

Neglecting the through space dipolar interaction between the magnetic moment of ^{195}Pt and the spin population located on the vanadium ion, which based on a Pt···V distance of 0.28 nm is of the order of 0.8 MHz, the following values are obtained:

1: $\rho_s \approx 0.17\%$ $\quad \rho_d \approx 2.6\%$

2: $\rho_s \approx 0.15\%$ $\quad \rho_d \approx 2.6\%$

leading to an overall estimate of the spin delocalization on the Pt atom of the order of 2.7% for both complexes. The order of magnitude is consistent with the value estimated from Löwdin population analysis (see main text).

T_1 and T_m vs temperature data

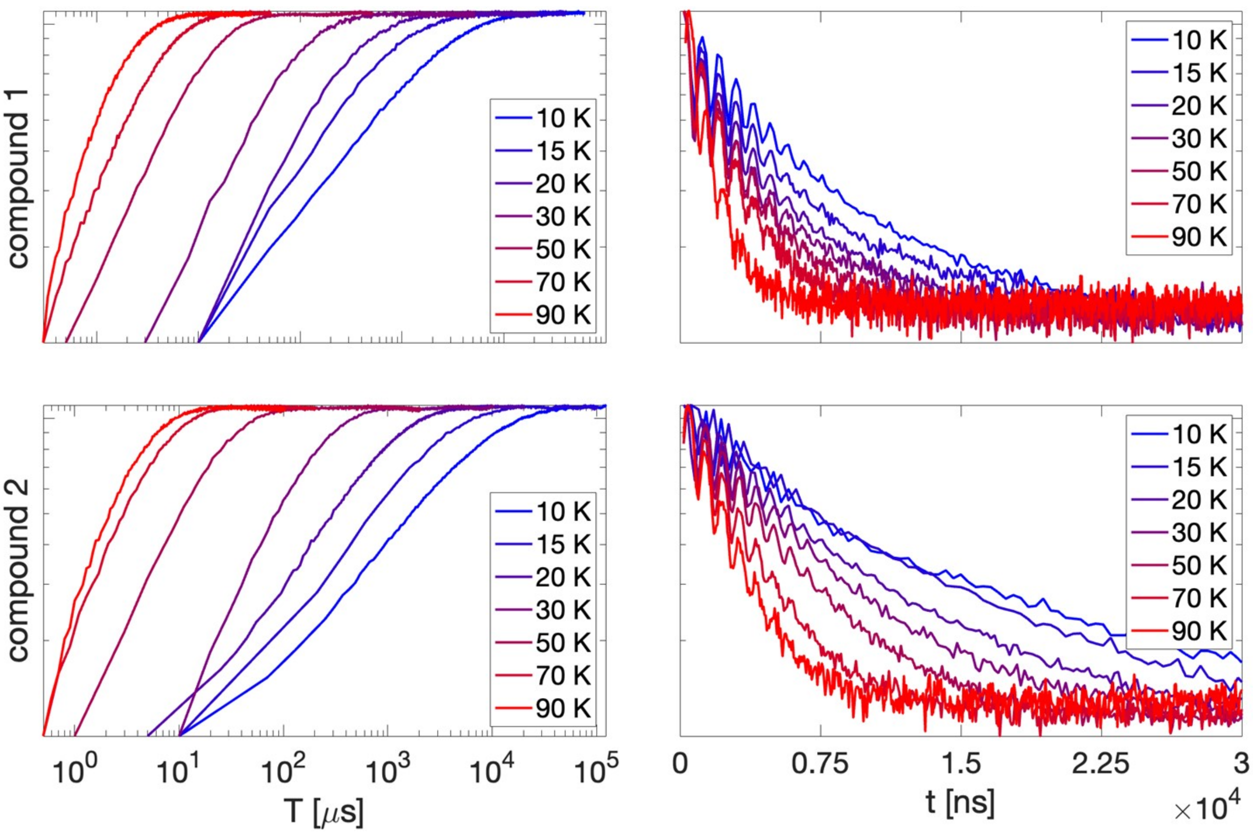

Fig. S9. Inversion recovery (left hand side) and echo decay (right hand side) traces for **1** and **2**.

Table S2. T_1 and T_m fitting parameters for **1** and **2**.

		T_m				T_1			
	Temp [K]	a_slow	t_slow [ns]	a_fast	t_fast [ns]	a_slow	t_slow [μs]	a_fast	t_fast [μs]
**1**	10	37109	6132	14536	169	27029	3959	28040	661
	15	20226	4935	12137	174	11481	1232	13830	233
	20	61340	4396	46680	191	16567	506	19441	116
	30	96734	3853	94174	230	73515	139	108426	42
	50	156308	3039	109834	201	729040	13	352467	4,8
	70	167546	2338	101956	183	620162	4,3	269841	1,8
	90	878308	994	–	–	1077160	1,76	515494	0,771
**2**	10	466	11481	215	2966	23594	11199	19785	1800
	15	21773	10420	26468	6800	35886	3498	26465	585
	20	73920	7307	16234	166	56231	1185	35777	286
	30	54954	5839	50209	5763	3066	869	227505	176
	50	191777	4532	97718	4513	272605	25	144054	9,6
	70	313254	3023	–	–	186083	9	379046	3,8
	90	237917	2105	–	–	403920	3,564	64279	1,106

Table S3. Best-fit parameters in eqn (2) for **1** and **2**.

	A_{dir} [Hz]	A_{loc} [MHz]	$\hbar\omega_{loc}$ [cm^{-1}]
**1**	17.4	1.7	109
**2**	8.6	4.5	177

Computational Procedures

Computational details. All the calculations were performed with the ORCA 4.2.1 quantum chemistry package[13] using (unless otherwise stated) the PBE0 hybrid exchange-correlation functional[14] along with D3 atom pairwise dispersion corrections.[15] The X-ray structures of **1** and **2** were optimized in the gas-phase by DFT. Def2-TZVPP basis sets were employed for all the atoms.[16] For platinum, we used an effective core potential to describe the inner core electrons.[17] For the optimization runs, 'TightOpt' convergence criteria were set. Afterwards, we ran analytical frequency calculations on the final optimized structures to verify that the geometry is a true local minimum of the potential energy surface.

For the calculation of the Spin Hamiltonian parameters (*g*-tensor and hyperfine couplings) on the final optimized structures by DFT, Douglas-Kroll-Hess Hamiltonian was applied to account for scalar relativistic effect, and a DKH-def2-TZVPP all-electron basis set[16] was employed for Pt. The employment of uncontracted or larger basis sets, such as quadruple-zeta basis, did not improve the results or led to worse agreement with experimental data.

The intermolecular exchange interactions were computed in dimer models. The models were made using two molecular neighboring units extracted from the crystal structure without any structural optimization, as shown in Fig. S2. For this set of calculations, the B3LYP functional was chosen[18,19] with def2-TZVPP basis for all atoms.[16] The same procedure was employed to compute the interdimer interaction, but in this case the two nearest neighboring monomers belonging to different dimers were extracted. The isotropic magnetic-coupling constants (*J*) were extracted within the broken symmetry (BS) approach,[20] as developed by Noodleman. Such a method allows the calculation of magnetic interactions by DFT without the necessity to employ computationally expensive multiconfigurational approaches. Therefore, single-point calculations on both the triplet and the BS state were performed. Afterwards, we mapped the spin states on the Heisenberg-Dirac-Van Vleck Hamiltonian $\hat{H}_{HDVV} = J\hat{\mathbf{S}}_1 \cdot \hat{\mathbf{S}}_2$ where $S_1 = S_2 = 1/2$, and computed the value of *J* with the following formula:

$$J = (E_{HS} - E_{BS})/(2S_1S_2) \qquad \text{(S3)}$$

In eqn (S3), E_{HS} is the energy of the high-spin (HS) triplet state (total spin $S = 1$) and E_{BS} is the energy of the BS state (non totally symmetric $S = 0$ singlet).

Model $\mathbf{1}_\delta$ was built starting from the coordinates of V, Pt, S, and O atoms of the optimized structure of **1**, but replacing the bridging thioacetates with terminal acetato ligands and adding protons as required to ensure charge neutrality (see Fig. S11).

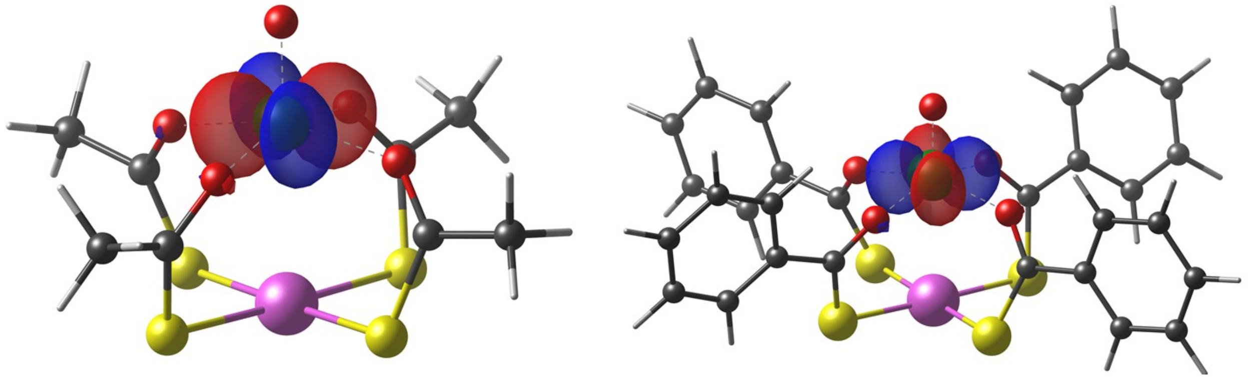

Fig. S10. DFT computed singly occupied Quasi-Restricted Orbital (QRO) of **1** (left) and **2** (right) with an isosurface value set to 0.05 $e^- a_o^{-3}$. Color code is the same as in Fig. S1.

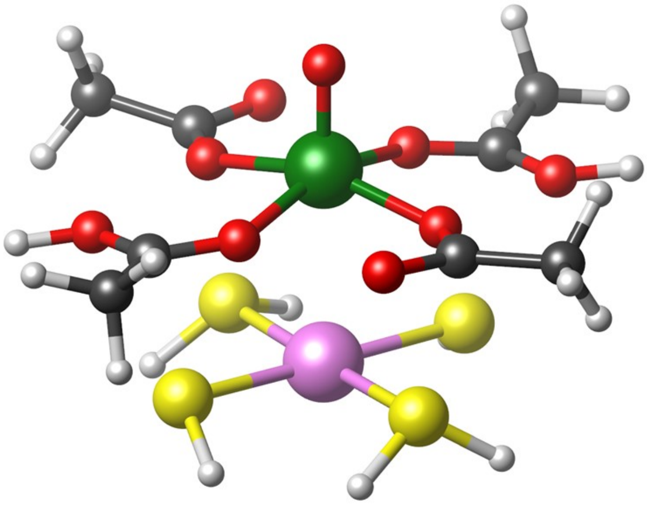

Fig. S11. Geometrical model $\mathbf{1}_{\boldsymbol{\delta}}$ employed to compute the *π* and *δ* contributions to the Fermi contact term. Color code is the same as in Fig. S1.

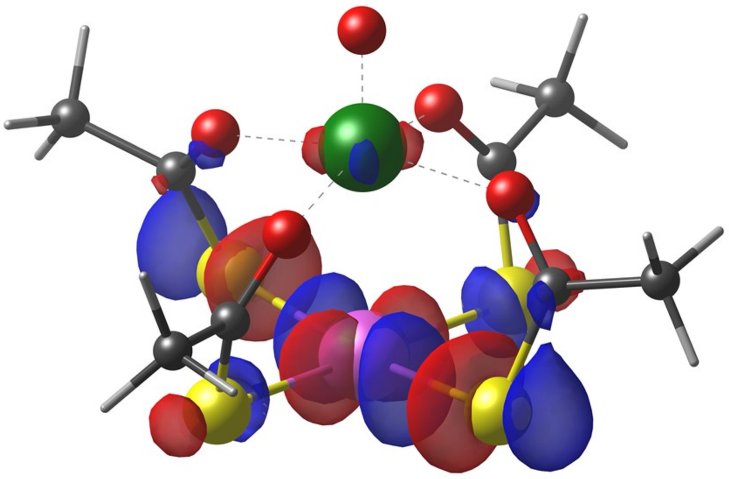

Fig. S12. Platinum-centered singly occupied Quasi-Restricted Orbital (QRO) of $\mathbf{1}^{-}$, as computed by DFT, showing that the anion is a $Pt^{I}V^{IV}$ species. The isosurface value is set to 0.05 e^{-} a_o^{-3} and the color code is the same as in Fig. S1. The alternative $Pt^{II}V^{III}$ configuration failed to converge to a different energy minimum and collapsed to the $Pt^{I}V^{IV}$ configuration.

Table S4. Main structural parameters of the optimized structures of **1** and **2** at the DFT level of theory.

	**1**	**2**
Pt-V distance	2.876 Å	2.848 Å
V-O1 bond length	2.010 Å	1.989 Å
V-O2 bond length	2.004 Å	2.012 Å
V-O3 bond length	2.015 Å	2.017 Å
V-O4 bond length	2.001 Å	1.984 Å
O1-V-Pt-S1 dihedral	19.907°	21.531°
O2-V-Pt-S2 dihedral	20.020°	22.317°
O3-V-Pt-S3 dihedral	20.108°	21.961°
O4-V-Pt-S4 dihedral	19.982°	21.585°
V-O1-S1-Pt dihedral	22.640°	24.388°
V-O2-S2-Pt dihedral	22.733°	25.085°
V-O3-S3-Pt dihedral	22.754°	24.694°
V-O4-S4-Pt dihedral	22.723°	24.582°

Table S5. Contributions to the ^{195}Pt hyperfine coupling tensor for **1**, **2**, and **1$_\delta$** as computed by DFT.[a]

**1**	$^{Pt}A_x$	$^{Pt}A_y$	$^{Pt}A_z$	*Iso*
Fermi contact	–19.8434	–19.8434	–19.8434	–19.8434
spin-dipole	3.0191	3.0077	–6.0268	-
spin-orbit	2.4922	2.5890	–15.8700	–3.5965
Total	–14.3321	–14.2468	–41.7407	–23.4399

**2**	$^{Pt}A_x$	$^{Pt}A_y$	$^{Pt}A_z$	*Iso*
Fermi contact	–16.9601	–16.9601	–16.9601	–16.9601
spin-dipole	2.8965	2.3397	–5.2362	-
spin-orbit	2.4678	1.9952	–14.6601	-3.3990
Total	–11.5959	–12.6252	–36.8564	–20.3592

**1$_\delta$**	$^{Pt}A_x$	$^{Pt}A_y$	$^{Pt}A_z$	*Iso*
Fermi contact	–5.6376	–5.6376	–5.6376	–5.6376
spin-dipole	–1.6403	–0.9894	2.6296	-
spin-orbit	–0.5102	–1.0304	0.2193	–0.4404
Total	–7.7881	–7.6574	–2.7887	–6.0780

[a] All values are in MHz.

Table S6. Calculated vibrational frequencies for **1**. The frequencies in bold are those having totally symmetric character within approximate C_4 point group symmetry.

Normal Mode	Frequency (cm^{-1})
6	60.82
7	61.28
8	62.04
9	**69.08**
10	**103.65**
11	107.87
12	108.15
13	112.45
14	116.42
15	119.92
16	124.84
17	128.02
18	**140.60**
19	164.95
20	168.43
21	170.69
22	184.71
23	188.33
24	198.18

Table S7. Calculated vibrational frequencies for **2**. The frequencies in bold are those having totally symmetric character within approximate C_4 point group symmetry. The normal modes below 60 cm^{-1} are mainly localized on the phenyl rings.

Normal Mode	Frequency (cm^{-1})
6	7.64
7	8.03
8	11.97
9	14.80
10	**15.34**
11	30.15
12	31.93
13	**35.72**
14	43.75
15	**68.83**
16	77.41
17	80.97
18	**96.08**
19	106.46
20	117.70
21	124.44
22	125.44
23	128.22
24	131.45
25	**135.07**
26	155.89
27	168.43
28	**170.61**
29	171.92
30	175.07
31	194.46
32	**196.90**

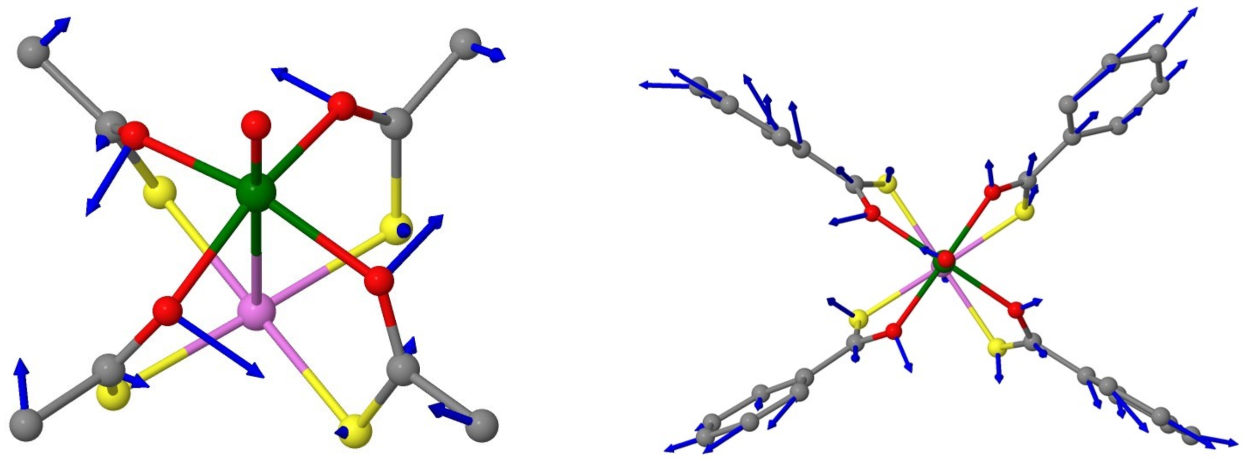

Fig. S13. Displacement vectors of the 10th normal mode of **1** at 104 cm^{-1} (left) and of the 28th normal mode of **2** at 171 cm^{-1} (right). Color code is the same as in Fig. S1. Hydrogen atoms are omitted for the sake of clarity.

References

1 W. L. F. Armarego, *Purification of laboratory chemicals*, Butterworth-Heinemann, Oxford, UK, 8th edn., 2017.

2 A. Nicolini, R. Galavotti, A.-L. Barra, M. Borsari, M. Caleffi, G. Luo, G. Novitchi, K. Park, A. Ranieri, L. Rigamonti, F. Roncaglia, C. Train and A. Cornia, Filling the Gap in Extended Metal Atom Chains: Ferromagnetic Interactions in a Tetrairon(II) String Supported by Oligo-α-pyridylamido Ligands, *Inorg. Chem.*, 2018, **57**, 5438–5448.

3 S. A. Beach, J. L. Guillet, S. P. Lagueux, M. Perfetti, B. N. Livesay, M. P. Shores, J. W. Bacon, A. L. Rheingold, P. L. Arnold and L. H. Doerrer, Heterotrimetallic {LnOVPt} complexes with antiferromagnetic Ln–V coupling and magnetic memory, *Chem. Commun.*, 2020, **56**, 11062–11065.

4 *TopSpin 4.2.0*, Bruker AXS Inc., Madison, Wisconsin, USA, 2022.

5 A. Belèn Meneses, S. Antonello, M. C. Arévalo and F. Maran, Double-Layer Correction for Electron-Transfer Kinetics at Glassy Carbon and Mercury Electrodes in N,N-Dimethylformamide, *Electroanalysis*, 2006, **18**, 363–370.

6 S. Antonello, G. Arrigoni, T. Dainese, M. De Nardi, G. Parisio, L. Perotti, A. René, A. Venzo and F. Maran, Electron Transfer through 3D Monolayers on Au25 Clusters, *ACS Nano*, 2014, **8**, 2788–2795.

7 E. W. Dahl, F. G. Baddour, S. R. Fiedler, W. A. Hoffert, M. P. Shores, G. T. Yee, J.-P. Djukic, J. W. Bacon, A. L. Rheingold and L. H. Doerrer, Antiferromagnetic coupling across a tetrametallic unit through noncovalent interactions, *Chem. Sci.*, 2012, **3**, 602–609.

8 S. Bachmann, R. Neufeld, M. Dzemski and D. Stalke, New External Calibration Curves (ECCs) for the Estimation of Molecular Weights in Various Common NMR Solvents, *Chem. Eur. J.*, 2016, **22**, 8462–8465.

9 M. P. Crockett, H. Zhang, C. M. Thomas and J. A. Byers, Adding diffusion ordered NMR spectroscopy (DOSY) to the arsenal for characterizing paramagnetic complexes, *Chem. Commun.*, 2019, **55**, 14426–14429.

10 S. Bachmann, B. Gernert and D. Stalke, Solution structures of alkali metal cyclopentadienides in THF estimated by ECC-DOSY NMR-spectroscopy (incl. software), *Chem. Commun.*, 2016, **52**, 12861–12864.

11 G. A. Bain and J. F. Berry, Diamagnetic Corrections and Pascal's Constants, *J. Chem. Educ.*, 2008, **85**, 532–536.

12 J. R. Morton and K. F. Preston, Atomic parameters for paramagnetic resonance data, *J. Magn. Reson.*, 1978, **30**, 577–582.

13 F. Neese, Software update: the ORCA program system, version 4.0, *WIREs Comput. Mol. Sci.*, 2018, **8**, e1327.

14 C. Adamo and V. Barone, Toward reliable density functional methods without adjustable parameters: The PBE0 model, *J. Chem. Phys.*, 1999, **110**, 6158–6170.

15 S. Grimme, S. Ehrlich and L. Goerigk, Effect of the damping function in dispersion corrected density functional theory, *J. Comput. Chem.*, 2011, **32**, 1456–1465.

16 F. Weigend and R. Ahlrichs, Balanced basis sets of split valence, triple zeta valence and quadruple zeta valence quality for H to Rn: Design and assessment of accuracy, *Phys. Chem. Chem. Phys.*, 2005, **7**, 3297–3305.

17 D. Andrae, U. Häußermann, M. Dolg, H. Stoll and H. Preuß, Energy-adjusted ab initio pseudopotentials for the second and third row transition elements, *Theor. Chim. Acta*, 1990, **77**, 123–141.

18 A. D. Becke, Density-functional thermochemistry. III. The role of exact exchange, *J. Chem. Phys.*, 1993, **98**, 5648–5652.

19 P. J. Stephens, F. J. Devlin, C. F. Chabalowski and M. J. Frisch, Ab Initio Calculation of Vibrational Absorption and Circular Dichroism Spectra Using Density Functional Force Fields, *J. Phys. Chem.*, 1994, **98**, 11623–11627.

20 A. Bencini and F. Totti, A Few Comments on the Application of Density Functional Theory to the Calculation of the Magnetic Structure of Oligo-Nuclear Transition Metal Clusters, *J. Chem. Theory Comput.*, 2009, **5**, 144–154.
